# Methods for Detection and Mapping of Methylated and Hydroxymethylated Cytosine in DNA

**DOI:** 10.3390/biom14111346

**Published:** 2024-10-23

**Authors:** Olga Kisil, Alexander Sergeev, Anna Bacheva, Maria Zvereva

**Affiliations:** 1 Department of Chemistry, Lomonosov Moscow State University, Leninskie Gory 1/3, Moscow 119991, Russia; olvv@mail.ru (O.K.); anbach@belozersky.msu.ru (A.B.); zverevame@my.msu.ru (M.Z.); 2 Gause Institute of New Antibiotics, 11 B. Pirogovskaya Street, Moscow 119021, Russia; 3 Orekhovich Institute of Biomedical Chemistry, Pogodinskaya Street, 10/8, Moscow 119121, Russia

**Keywords:** DNA methylation, epigenetics, 5-hydroxymethylcytosine, oxidation, glycosylation, affinity enrichment, DNA-operating enzymes, HPLC, next-generation sequencing

## Abstract

The chemical modifications of DNA are of pivotal importance in the epigenetic regulation of cellular processes. Although the function of 5-methylcytosine (5mC) has been extensively investigated, the significance of 5-hydroxymethylcytosine (5hmC) has only recently been acknowledged. Conventional methods for the detection of DNA methylation frequently lack the capacity to distinguish between 5mC and 5hmC, resulting in the combined reporting of both. The growing importance of 5hmC has prompted the development of a multitude of methods for the qualitative and quantitative analysis of 5hmC in recent years, thereby facilitating researchers’ understanding of the mechanisms underlying the onset and progression of numerous diseases. This review covers both established and novel methods for the detection of cytosine modifications, including 5mC, 5hmC, 5-formylcytosine (5fC) and 5-carboxylcytosine (5caC), with a particular focus on those that allow for accurate mapping and detection, particularly with third-generation sequencing. The review aims to help researchers choose the most appropriate methods based on their specific research goals and budget.

## 1. Introduction

Six decades have elapsed since the discovery of 5-methylcytosine (5mC) in DNA. During this period, the pivotal function of 5mC as an epigenetic marker has been repeatedly substantiated. In essence, it constitutes a chemical modification that regulates gene expression in eukaryotic cells. Maintaining the distribution of 5mC across the genome and the degree of DNA methylation through a balancing of methylation and demethylation processes is essential to the normal functioning of living cells. Atypical DNA methylation has been identified as a contributing factor in a range of human diseases, with the dynamic status of methylation influencing various physiological processes, including genome stability, embryogenesis, genomic imprinting and X-chromosome inactivation [1]. Hypermethylation can result in the silencing of tumor suppressor genes, thereby promoting cancer development, while hypomethylation can lead to the activation of oncogenes [2]. Neurological conditions such as Rett syndrome and Fragile X syndrome are associated with aberrant methylation, which can result in impaired brain function and developmental delays [3]. Aberrant methylation may also play a role in autoimmune diseases such as systemic lupus erythematosus, type 1 diabetes, rheumatoid arthritis, Graves’ disease, and Hashimoto’s disease [4].

De novo DNA methyltransferases DNMT3a and DNMT3b establish the methylation pattern in unmethylated embryonic DNA. This is then maintained during subsequent cell divisions by DNMT1, based on the methylation pattern of the template strand during replication. DNA methyltransferases transfer a methyl group from the donor S-adenosyl-L-methionine to the fifth position of a cytosine residue in CpG dinucleotides, thereby establishing the pattern of cytosine methylation. This pattern is reproduced by DNMT1, which is associated with the replication fork. A total of 60–80% of the roughly 28 million CpGs in the human genome are methylated [1]. The majority of CpGs in the mammalian genome are diffusely distributed, while a small fraction exists in CpG islands (CGIs) where the density of CpG dinucleotides is greatly elevated. CGIs are, on average, 1000-base pair (bp)-long and often overlap with annotated gene promoters. CGIs are usually unmethylated, especially in germ cells and early embryos, but some of them become methylated during normal development, causing stable promoter silencing [5]. A few CGIs are methylated in normal tissues and may play a role in the regulation of the expression of tissue-specific genes [6].

**Figure 1:**
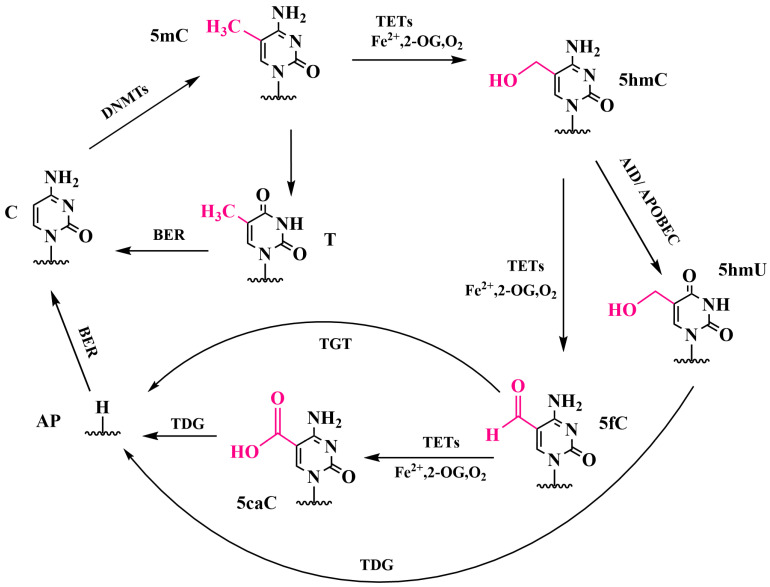
Reaction scheme of active demethylation of 5-methylcytosine in DNA. Modified cytosine groups are shown in red. AID/APOBEC are enzymes of the cytidine deaminase group that induce mutations in DNA and RNA due to their ability to deaminate cytidine to uridine. Figure based on data from [11,12,13].

The activity of DNA methyltransferases is influenced by a number of factors, including the recognition of histone modifications, the recruitment of the enzymes by transcription factors, and their interaction with elements present in the flanking sequences [7]. It is well documented that DNA methyltransferases play a crucial role in embryogenesis; moreover, the loss of function of these enzymes is lethal [8]. The reverse process, demethylation, can occur in two ways: passively, through the loss of maintenance during cell division, or actively. Active DNA demethylation in mammals occurs via the sequential oxidation of 5mC by ten-eleven translocation (TET) proteins [9], resulting in three intermediate products: 5-hydroxymethylcytosine (5hmC), 5-formylcytosine (5fC) and 5-carboxylcytosine (5caC) (Figure 1) [10].

The products of further oxidation of 5hmC by TET proteins, 5fC and 5caC, can subsequently be recognized and cleaved by thymine-DNA glycosylase (TDG), thereby facilitating the restoration of unmethylated cytosine through the base excision repair (BER) pathway [14]. 5fC and 5caC are transient intermediates in the DNA demethylation pathway. It has been demonstrated that the presence of 5fC and 5caC in DNA recognition sites enables the recruitment of specific proteins to perform specific functions. Consequently, 5fC and 5caC may serve a purpose beyond that of mere intermediates in the DNA demethylation pathway [15]. The impact of all identified cytosine modifications on the rate of RNA polymerase II (Pol II) nucleotide incorporation and substrate specificity for DNA templates containing a site-specific C, 5mC, 5hmC, 5fC or 5caC was examined [16,17]. It was demonstrated that the Pol II polymerization rates and specificity constants for GTP incorporation against 5fC and 5caC are significantly reduced in comparison with those for a C template. In contrast, no notable changes were observed for the 5mC and 5hmC templates. The discrimination of GTP over ATP was reduced by a factor of ~30 for the 5fC template in comparison with the C template. The authors suggest that Pol II pausing at the 5fC and 5caC sites may serve as a signal for the recruitment of TDG and BER machinery to the sites. It has been demonstrated that oxidized 5mC derivatives can induce mutation, with 5caC being the most mutagenic. However, no impact on replication efficiency was observed in *Escherichia coli* [16,17].

5hmC is a relatively stable and tissue-specific modification. For example, a fairly high content of 5hmC has been demonstrated in tissues of the central nervous system [18]. The first identification of 5hmC as a naturally occurring DNA base was made in bacteriophages in the 1950s [19]. In the 1970s, 5hmC was identified in DNA extracted from the brains and livers of rats [20]. In the 2000s, the first functional data on the hydroxylation of bases in DNA were obtained, specifically the in vitro inhibition of the interaction between DNA and proteins containing a methyl-CpG-binding domain [21]. This indicated a potential mechanism through which this modification might affect gene expression and chromatin structure.

The significance of 5hmC in the functioning of the human genome changed dramatically in 2009. Two studies published in the journal *Science* reported the presence of significant amounts (up to 0.6% of the total cytosine content) of this modified base in Purkinje cells of the cerebellum and in embryonic stem cells of animals. They also identified the enzyme responsible for generating the modification [9,22].

In adult mammals, the level of 5hmC is highly variable but is consistently detected in all tissues and cell types that have been studied thus far [23]. It is notable that the distribution of 5hmC across the genome differs from that of 5mC [24]. 5hmC is localized in enhancers, promoter CGIs and intragenic regions (“bodies”) of genes. Furthermore, 5hmC content in enhancer sequences is directly correlated with enhancer activity. In CGIs, 5hmC is essential to maintaining promoters in an unmethylated state, while in the gene body, 5hmC is believed to inhibit the initiation of antisense transcription [25,26].

In the adult human brain, 5mC is predominantly found within gene bodies, whereas 5hmC is found in promoter regions. 5hmC is usually associated with increased gene expression, consistent with its co-localization with euchromatin [27]. In the brain, 5hmC levels are low in stem cells and high in fully differentiated neurons. The quantity of 5hmC differs among brain cell types, with 0.6% of all nucleotides being present in Purkinje neurons and 0.2% in granular cells [22]. Gross and colleagues definitively characterized 5hmC in the human prefrontal cortex and showed that approximately 8% of all CpG sites on autosomal chromosomes contain 5hmC [26]. The density of 5hmC is the highest in enhancer regions and within exons.

The enrichment of 5hmC in DNA regions encoding genes that regulate embryonic development, both in transcribed and promoter regions, proves that 5hmC plays a significant role in cellular differentiation and epigenetic regulation [28,29]. Given that the DNA methyltransferase DNMT1 has a low affinity for binding to 5hmC [30], it seems probable that 5hmC contributes to the exclusion of DNMT1 from methylating cytosines. This inhibits maintenance methylation and promotes DNA demethylation during differential cell division.

It is noteworthy that the level of 5hmC decreases as embryonic stem cells differentiate. This suggests that 5hmC plays a specific role in this process. 5hmC is now being recognized as a biomarker in disease diagnostics, just as 5mC was before it (see Section 8) [31,32,33,34,35,36,37,38]. Potentially, 5hmC could be employed as a means of identifying aggressive subtypes of cancer that are related to cancer stem cells. TET1, which is responsible for the sequential oxidation of 5mC to 5hmC, has been demonstrated to regulate stem cell properties and the cell cycle of cancer stem cells in triple-negative breast cancer via DNA demethylation [39].

The identification of 5hmC in DNA and the subsequent discovery of the enzyme responsible for its formation have raised questions about its functional role, leading to the development of highly effective methods for the detection of 5hmC in DNA. Over the past decade, significant progress has been made in the development of analytical techniques for the quantitative determination of 5hmC. The growing interest in 5hmC has acted as a catalyst for the development of several methods for the analysis of its distribution throughout the genome.

The existing methods for characterizing the methylation status are typically adapted to develop methods to assess the degree of hydroxymethylation in DNA samples. The standard, widely used, and commercialized methods for DNA methylation analysis, both at the level of individual genes and across the whole genome, can be divided into the following groups: 1. high-performance liquid chromatography with mass spectrometry (HPLC-MS); 2. methods based on the interaction of 5mC with methyl-CpG-binding proteins or antibodies against 5mC (MBD-seq and MeDIP-seq); 3. methods based on the enzymatic activity of methylation-sensitive restriction endonucleases; 4. methods based on sodium bisulfite conversion; 5. next-generation sequencing (NGS) and third-generation sequencing (TGS). SMRT and Nanopore are the best options.

It is evident that only a few of these methods can be used directly. This is because 5hmC is present in biological samples at lower levels than the detection limits of many of these methods. Furthermore, 5hmC has a structure similar to 5mC and C, which affects the accuracy of the analysis. Methods for analyzing 5hmC must be highly sensitive and have excellent specificity to eliminate background interference from 5mC and C. In order to map 5hmC to specific positions in the genome, methods based on the additional labeling and enrichment of 5hmC have been developed [40].

In this review, we discuss methods for determining the degree of hydroxymethylation in DNA samples from a chemical perspective. We group the methods according to the chemical transformation of 5hmC, which is the first step of the protocol. The following sections of the review set out the details of each method and provide a clear analysis of its advantages and disadvantages. It is evident that no single method is universally applicable. However, by grasping its underlying principles and the insights it offers, researchers can select the most appropriate method for determining hydroxymethylation levels, tailored to their specific research requirements. This review focuses on the most well-characterized, reliable, and accessible methods for determining hydroxymethylation levels which we have identified as being the most suitable for researchers.

## 2. Standard Methods for DNA Methylation Analysis

### 2.1. High-Performance Liquid Chromatography with Mass Spectrometric Detection (HPLC-MS) and Other Chromatographic Methods

In mammalian cells, 5hmC was first detected by thin-layer chromatography (TLC) and then confirmed quantitatively by the HPLC and MS methods. In 2009, Kriaucionis et al. observed a decrease in the amount of 5mC in DNA from Purkinje cells and the presence of an unidentified spot on TLC plates while determining the total cytosine methylation in CGIs [22]. The authors hypothesized that this was the 5-hydroxymethyl-2′-deoxycytidine (5hmC) monophosphate found in the DNA of T4 bacteriophages. In fact, the commercial 5hmC monophosphate was shown to form a spot that matched the unidentified spot. The analysis of genomic DNA by reversed-phase high-performance liquid chromatography (RP-HPLC) revealed the presence of a small but reproducible peak at the same position as the 5hmC peak in synthetic DNA hydrolysate. To prove conclusively that the unidentified spot was 5hmC, the authors analyzed the corresponding fraction by using high-resolution mass spectrometry (MS). The mass spectra revealed the presence of two ions with mass-to-charge ratios (*m*/*z*) of 142.06 ± 0.01 and 280.11 ± 0.02, corresponding to the theoretical isotopic molecular masses of ions derived from 5hmC with *m*/*z* 142.06 and 280.09, respectively. The corresponding 5hmC fraction from the synthetic DNA produced the same ions. It was shown that 5hmC constituted 0.6% of the total nucleotides in Purkinje cells and 0.2% in granular cells and was absent in cancer cell lines. In the same year, Tahiliani et al. used TLC analysis to show that in cells overexpressing TET1, TET1 could catalyze the conversion of 5mC into another modified base [9]. By using MS, the authors identified this newly discovered nucleotide as 5hmC. To date, only Tet family proteins have been shown to generate 5hmC from 5mC in mammalian genomic DNA.

The unexpected presence of 5hmC in DNA and the discovery of the enzyme that facilitates its formation raised questions about its functional role and led to the development of methods to detect 5hmC in DNA. Over the past decade, significant progress has been made in the development of analytical methods for the quantitative determination of 5hmC. Currently, both TLC and HPLC combined with mass spectrometric detection (HPLC-MS) are used to detect 5hmC in DNA. The advantage of TLC is its low cost and simplicity, but it requires radioactive substrates, and its accuracy and detection limits are not comparable to other available methods. The identification of 5hmC by chromatography is frequently complicated by the co-elution of other DNA and RNA nucleotides that may be present in biological samples [41]. The hydrolysis of DNA with formic acid to nitrogenous bases requires complete removal of RNA, as RNA is rich in modified bases, which can interfere with the accurate quantitative assessment of DNA methylation [42].

Alternatively, DNA can be enzymatically hydrolyzed prior to HPLC. Typically, DNAse I is used in combination with nuclease P1 to generate mononucleotides, followed by dephosphorylation with alkaline phosphatase; a comprehensive overview of enzymes and their combinations used in the digestion of genomic DNA was compiled by Lai et al. [43]. While digestion reactions are usually carried out in solution, in 2018, Yin et al. developed a novel strategy for genomic DNA digestion based on capillary bioreactors containing immobilized enzymes [44]. Briefly, the DNA sample is passed through a series of bioreactors containing immobilized benzonase, snake venom phosphodiesterase and alkaline phosphatase, resulting in >99.5% digestion within 10 min. This sample preparation technique facilitated the detection of the oxidative stress biomarker 8-oxo-7,8-dihydro-2′-deoxyguanosine (8-oxodG) and 5hmC in DNA samples.

To determine the total (genome-wide) levels of 5mC and its oxidation products, the HPLC-MS method is widely used due to its inherent selectivity and sensitivity [45]. The quantitative detection of 5hmC by HPLC-MS or HPLC-MS/MS has been performed by several research groups. A method using HPLC-MS/MS in electrospray ionization (ESI) mode (HPLC-ESI-MS/MS) has been developed for the quantitative determination of 5mC, 5hmC, 5-formyl-2′-deoxycytidine (5fC) and 5-carboxyl-2′-deoxycytidine (5caC) in genomic DNA from mammalian tissues and cells [10].

B. Vetö et al. employed formic acid to hydrolyze DNA to nucleobases and subsequently detected 5mC and 5hmC levels in the hydrolysate of genome DNA by LC-MS/MS [46]. The method facilitated the characterization of DNA methylation and hydroxymethylation levels in mouse tissues and cell lines. Additionally, it was observed that the inhibition of DNA methyltransferase not only resulted in a reduction in 5mC content but also led to an increase in 5hmC levels in hematopoietic cell lines.

In a separate study, Le and colleagues demonstrated the feasibility of using HPLC-ESI-MS/MS with multiple reaction monitoring (HPLC-ESI-MS/MS-MRM) for the simultaneous measurement of 5mC and 5hmC levels in DNA enzymatic hydrolysates [41]. The HPLC-ESI-MS/MS-MRM method allows for the quantitative determination of 5hmC, 5mC and C with high reproducibility and low detection limits (approximately 0.5 fmol per sample). This detection limit corresponds to 50 ng of genomic DNA hydrolysate sufficient for measuring 5hmC levels at 0.1%. In addition, the method is relatively rapid, requiring only a few hours to a day after genomic DNA extraction: DNA hydrolysis to nucleoside components takes 1–2 h, and the measurement of 5hmC and 5mC levels by using the HPLC-ESI-MS/MS-MRM method takes 6 min per sample. A readily ionizable fragment can be introduced to improve ESI efficiency. Chemical derivatization has been shown to improve separation by liquid chromatography and increase detection sensitivity for all four cytosine modifications (5mdC, 5hmdC, 5fdC and 5cadC) that occur in DNA during demethylation.

The analysis of global DNA methylation by HPLC-MS/MS is characterized by high precision, sensitivity and reproducibility and is performed across the entire genome, regardless of site or sequence. DNA methylation levels can be determined with unprecedented accuracy. For mammalian DNA, where ~2–5% of all cytosine residues are methylated, the HPLC-MS/MS method has been validated for the determination of methylation levels in the range of 0.05–10% and allows for the accurate detection of differences between samples down to ~0.25% of total cytosine residues, corresponding to ~5% of the differences in global DNA methylation. Typically, 50–100 ng of DNA sample is required for HPLC-MS/MS, although much smaller amounts (as little as 5 ng) have been successfully profiled [47,48]. An important advantage of this method is that it is not affected by DNA sample quality (e.g., DNA from formalin-fixed, paraffin-embedded samples can be analyzed).

#### 2.1.1. HPLC-MS/MS with Stable Isotope Labeling

**Figure 2:**
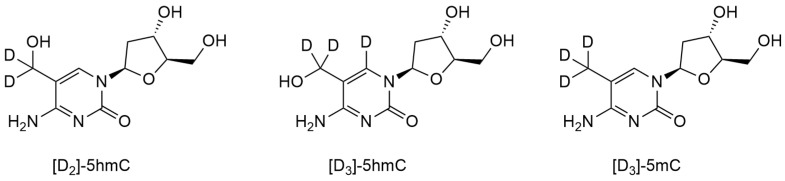
Internal standards in HPLC-MS/MS with stable isotope labeling used for the quantitative assessment of 5mC, 5hmC, 5fC and 5caC levels. The figure is based on data from the papers [49,50].

Du et al. used HPLC-MS/MS for the quantitative assessment of 5mC, 5hmC, 5fC and 5caC in both tumor and non-tumor regions of gastric cancer [49]. Calibration curves were constructed by measuring the quantities of internal standards with the addition of [D_2_]-5hmC (Figure 2). The HPLC-MS/MS analysis revealed that the level of 5hmC was significantly reduced in tumor regions. The level of 5mC was also moderately decreased in tumors, while 5fC and 5caC were barely detectable. The HPLC-MS method minimizes potential cross-interference between the measurements of molecules with low levels of modified bases.

Because of its advantages of sterility, availability in large volumes and noninvasiveness for the patient, urine is a preferred diagnostic biofluid for analysis. Yin et al. developed a sensitive and specific assay for the quantitative determination of 5hmC in human urine [50]. Urine samples were desalted and enriched by using automated solid-phase extraction with the addition of the stable isotopes [D_3_]-5hmC and [D_3_]-5mC (Figure 2), followed by HPLC-MS/MS analysis. The extraction efficiency of this method was found to be approximately 100% for 5hmC and 70–90% for 5mC. In an analysis of 13 volunteers using the developed method, the authors demonstrated for the first time the presence of 5hmC in human urine: the level of 5hmC (22.6 ± 13.7 nmol/L) was comparable to the level of its precursor, 5mC (52.4 ± 50.2 nmol/L).

Münzel et al. developed a quantitative HPLC-MS/MS method to investigate the distribution and relative amounts of 5hmC and 5mC in mammalian brain [18]. Nucleosides were synthesized in both natural and isotopically labeled [^18^O] and [^2^H] forms, and their levels were determined in various brain tissues of 90-day-old mice. The tissues were homogenized, and the DNA was extracted. Subsequently, the DNA was subjected to complete hydrolysis, isotopically labeled compounds were added, and the resulting nucleoside mixture was analyzed by HPLC-MS. In all experiments, 5hmC and 5mC were well separated and eluted with retention times of 12.3 and 18.5 min, respectively. In each experiment, one signal was recorded for the natural (light) and one for the synthetic (heavy) compounds. A quantitative assessment was performed by comparing the ion current integrals of the natural compound (as determined in the experiment) with the corresponding heavy-labeled derivatives (known amounts) by using a calibration curve. It was shown that 5hmC content is particularly high in brain tissues involved in higher cognitive functions. In 2013, Zhang et al. used a highly sensitive and accurate HPLC-MS/MS method using the isotopes ^13^C and ^15^N to precisely determine the levels of 5hmC and 5mC in colorectal cancer and showed that 5hmC levels are reduced by approximately six times in tumors compared with adjacent normal tissue [51]. An analysis of 5hmC levels and the 5hmC:5mC ratio during tumor progression may provide insights into the role of this modification in cellular immortalization and transformation.

#### 2.1.2. Selective Derivatization of 5hmC Prior to HPLC

**Figure 3:**
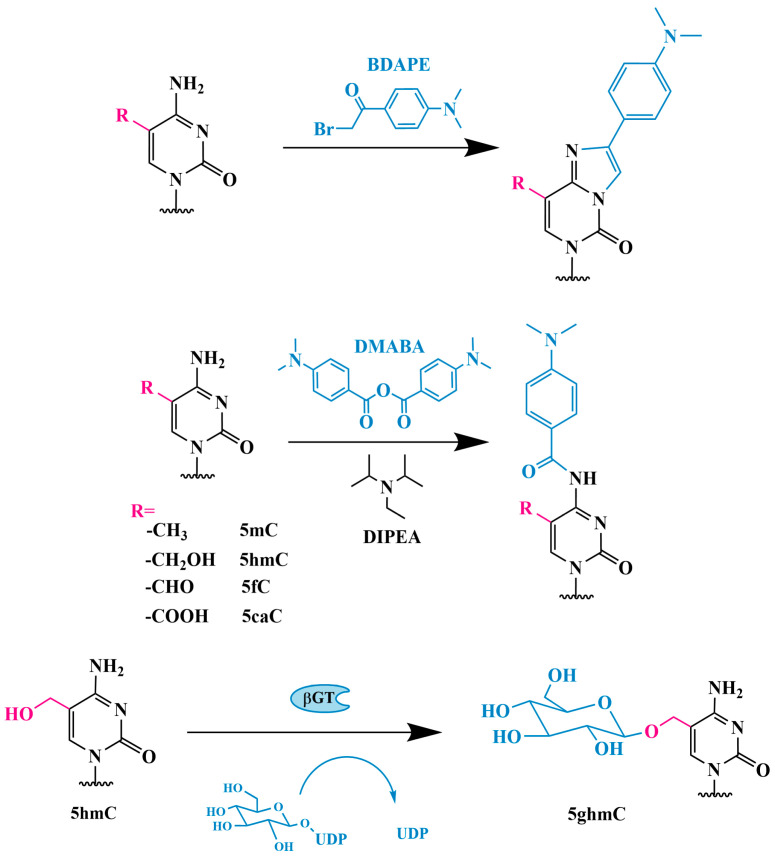
Chemical derivatization of cytosine modifications prior to HPLC with the following reagents: top panel—2-bromo-1-(4-dimethylaminophenyl)ethanone (BDAPE); middle panel—4-dimethylaminobenzoyl anhydride (DMABA); bottom panel—transfer of a glycosyl group using T4 β-glycosyltransferase (βGT) to form β-glycosyl-5-hydroxymethyl-2′-deoxycytidine (5gmC). The figure is based on data from the papers [54,55,56].

HPLC-ESI-MS/MS is currently the only known method for the quantitative determination of not only 5hmC but also 5fC and 5caC. The abundance of 5mC and its oxidation products varies within the genome: 5hmC is approximately 10–100 times less abundant than 5mC, while 5fC and 5caC are approximately ~40–1000 times less abundant than 5hmC [52,53]. The presence of a large number of unmodified nucleosides, in addition to the poor ionization of 5hmC, 5mC, 5fC and 5caC, often leads to low sensitivity in HPLC-ESI-MS/MS analysis. To overcome this problem, Tang et al. proposed the selective derivatization of cytosine fragments by using 2-bromo-1-(4-dimethylaminophenyl)ethanone (BDAPE) in conjunction with HPLC-ESI-MS/MS for the simultaneous determination of all these cytosine modifications in genomic DNA (Figure 3) [54].

The chemical derivatization significantly increased the detection sensitivity (35–123-fold) and improved the retention behavior of these cytosine modifications in the HPLC-ESI-MS/MS analysis. The detection limits for the 5mC, 5hmC, 5fC and 5caC derivatives were 0.10, 0.06, 0.11 and 0.23 fmol, respectively. By using this method, the authors demonstrated significant depletion of 5hmC, 5fC and 5caC in human colorectal cancer tissues compared with adjacent normal tissues, which is consistent with the findings by Zhang et al. [51].

A similar method but using a different modifying reagent (4-dimethylaminobenzoyl anhydride; Figure 3) was used by Guo et al. [55]. The application of this reagent improved the separation and increased the sensitivity of detection for 5mC, 5hmC, 5fC and 5caC in genomic DNA extracted from both human breast cancer tissue and adjacent normal tissue.

Tang et al. developed a method to selectively transfer a glycosyl group to the hydroxymethyl portion of 5hmC (Figure 3), resulting in the formation of a more hydrophilic residue (β-glycosyl-5-hydroxymethyl-2′-deoxycytidine, 5gmC) by using T4 β-glycosyltransferase (βGT) [56]. The more hydrophilic 5gmC can be selectively enriched on an amino-silica column prior to HPLC-MS/MS analysis, significantly improving the sensitivity and accuracy of detection.

#### 2.1.3. 5hmC Quantitation Using Guanine as an Internal Standard

In 2021, Németh et al. described a novel quantitation method using guanine as an internal standard in HPLC-MS/MS for the detection of 5hmC and 5mC in DNA [57]. This approach demonstrated high accuracy and precision, detecting 5hmC at 0.005–0.5% and 5mC at 1–15%. The key advantage of this technique is that it does not require the use of an expensive internal isotope-labeled standard, as the molar ratio of G and C in DNA samples is equal. The method demonstrated efficacy in identifying 5mC and 5hmC concentrations in a range of samples, with as little as 500 ng of DNA being sufficient for the detection of 5hmC at a level of 0.005%.

To date, HPLC-MS remains the most accurate method for the quantitative determination of total 5hmC in biological samples due to its high sensitivity and selectivity. The use of HPLC-MS for the determination of hydroxymethylation levels in DNA samples has been extensively described in the literature [58]. However, this method is technically complex and requires the use of expensive equipment. Developing a mass spectrometry protocol requires experience with specific sample handling techniques and instrument calibration for accurate results. Other drawbacks include the need for a significant amount of DNA and, most importantly, the inability to provide mapping information, i.e., the distribution of 5hmC across the DNA sequence. HPLC-MS is not suitable for clinical analysis, where the rapid assessment of changes in 5hmC levels and distribution at specific gene loci is required.

## 3. Chemical or Enzymatic Modification of DNA Containing 5hmC to Increase Detection Sensitivity Based on Various Detection Principles

### 3.1. Glycosylation

Glycosylation has already been mentioned as a preliminary chemical “labeling” of 5hmC to increase the sensitivity of HPLC-MS [56]. Bacteriophage T4 β-glycosyltransferase (βGT) can transfer a glucose fragment from uridine diphosphate glucose (UDP-Glc) to the hydroxyl group of 5hmC, forming β-glycosyl-5-hydroxymethylcytosine (5gmC) (Figure 3). When 5hmC is present in DNA, the UDP-Glc glycosidic group is transferred to the hydroxymethyl group of 5hmC by T4 βGT both in vivo and in vitro, regardless of the type of DNA, including the unglycosylated T4 genome. Neither C nor 5mC are substrates for T4 βGT and are not modified. It is only 5hmC in genomic DNA that can be covalently modified with a glycosidic group to form 5gmC, and βGT can glycosylate 5hmC regardless of the DNA sequence and structural context, making it an ideal candidate for this analysis [40].

#### 3.1.1. Glycosylation and Detection Using [^3^H]-Glucose

To increase the sensitivity of the quantitative determination of 5hmC in genomic DNA, Szwagierczak et al. used radiolabeled glucose [59]. The research study demonstrated that the incorporation of radiolabeled UDP-6-[^3^H]-glucose (D-glucose labeled with [^3^H] at the C6 atom) into DNA reflects the presence of 5hmC. The percentage of 5hmC relative to the total cytosine content was calculated from the incorporation of [^3^H]-glucose by using a calibration curve measured with a series of reference fragments for each experiment. The resulting curve was linear in the range of 0.25 to 2%, although this range begins at a relatively high 5hmC concentration which is significantly above the physiological 5hmC level. The authors emphasized that this method allows for the quantitative determination of 5hmC content in the genome with high sensitivity and allows for the simultaneous processing of a large number of samples without the need for specialized and expensive equipment. This method is useful for determining the global 5hmC content in genomic DNA, particularly in situations where limited tissue is available, such as in isolates of rare cell types and clinical samples.

#### 3.1.2. 5hmC Identification Using J-Binding Protein 1

The 5mC identification method based on the interaction of 5mC with methylated DNA-binding domains (MBDs), known as MBD-seq, cannot be used directly for mapping. Methyl-CpG-binding MBDs present in several chromatin-repressing proteins (e.g., MBD1 and MBD2) do not effectively recognize 5hmCpG [60,61]. The MBD2b/MBD3L1 complex is used, for example, to analyze CGI methylation patterns in mammals [62]. Testing the affinity of the MBD2b/MBD3L1 complex with 5mC and 5hmC on 76-mer oligonucleotides showed that the complex has negligible or no affinity for C76 or 5hmC76, suggesting that MBD2b and MBD3L1 form a protein complex that can only recognize 5mC in CpG sequences, but not 5hmC at the same sites. This characteristic of MBDs allows MBD-seq to be used exclusively for the detection of 5mC, with the presence of 5hmC in the sample having no impact on the result. The selective capture of 5hmC-containing DNA fractions can be achieved through the use of alternative proteins following a preliminary glycosylation step [63].

**Figure 4:**
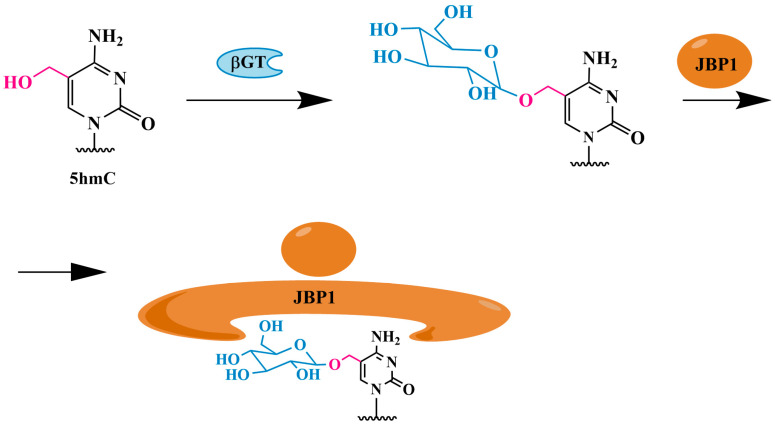
The method for the identification of 5hmC by glycosylation to 5gmC and binding by J-binding protein 1 (JBP1) immobilized on magnetic beads. The scheme is based on data from [63,64].

The 5hmC identification method using J-binding protein 1 (JBP1) immobilized on magnetic beads (Figure 4) involves the selective glycosylation of 5hmC residues by βGT to form 5gmC, which can be selectively captured by JBP1 bound to magnetic beads (JBP1 pulldown). The precipitated DNA was analyzed by quantitative polymerase chain reaction (PCR). The JBP1 pulldown approach was used to identify 5hmC in gene promoters in human embryonic stem cells [64].

This approach was later refined into the JBP1-seq method [65]. The recombinant JBP1 protein was expressed in a modified form with additional affinity tags, allowing for protein purification and biotinylation. Following purification, the biotinylated JBP1 was conjugated to streptavidin-coated magnetic beads. These modifications allow for a much more efficient “extraction” of the 5hmC-containing DNA fraction.

#### 3.1.3. Method of Sequential Glycosylation, Oxidation and Biotinylation (GLIB)

**Figure 5:**
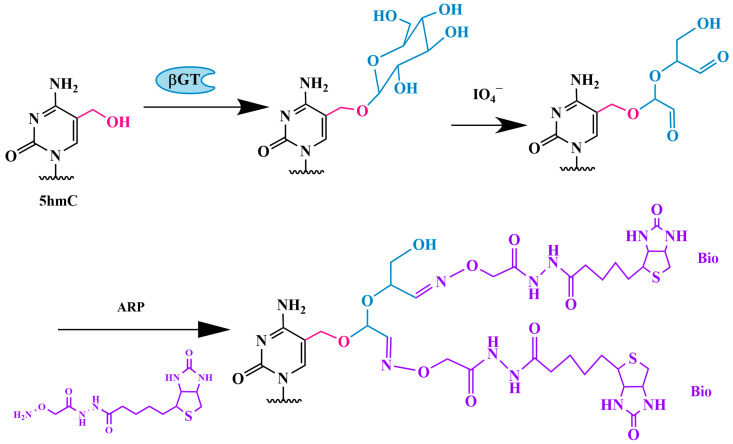
The GLIB method for the identification of 5hmC. It includes enzymatic glycosylation, periodate oxidation and biotinylation. The scheme is based on data from [66].

Another method for the detection of 5hmC by glycosylation is the GLIB method, which stands for glycosylation, periodate oxidation and biotinylation (Figure 5). The protocol consists of the following steps: 1. the glycosylation of 5hmC residues to 5gmC; 2. the oxidation of the glucose residue with sodium periodate, which converts vicinal hydroxyl groups into aldehyde groups, forming two aldehyde groups and releasing formate; 3. the addition of two molecules of an aldehyde-reactive biotin-containing probe (ARP), resulting in the attachment of two molecules of biotin (Bio) to each modified residue.

The biotin-containing genomic DNA fragments are then isolated by using streptavidin-coated magnetic beads, eluted and subjected to sequencing. This method allows for the quantitative labeling and precipitation of DNA fragments containing only a single 5hmC residue. The sample preparation and GLIB can be completed in 2–3 days [66].

**Figure 6:**
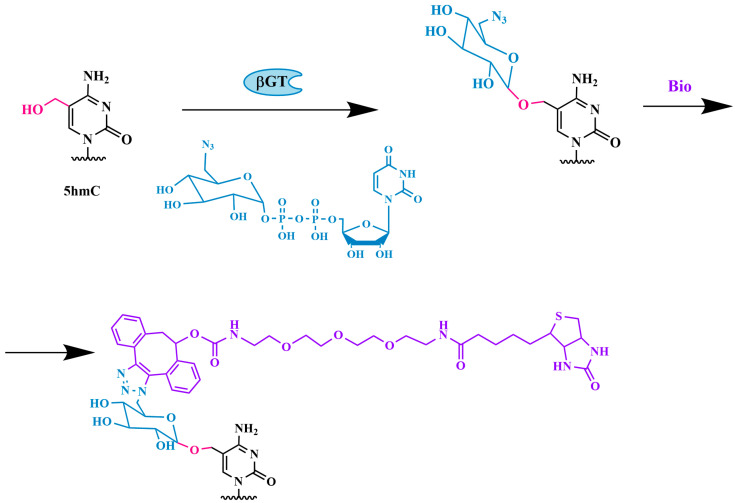
A method for the identification of 5hmC similar to GLIB. It includes enzymatic glycosylation with azide-containing glucose derivative with subsequent biotinylation. The scheme is based on data from [67].

A similar strategy was used by Song et al. [67]. A specially synthesized fragment, UDP-6-N3-glucose, which contains an azide group at the C6 position of glucose, was transferred to 5hmC by using βGT (Figure 6). The azide group then reacts with a fluorescently labeled alkyne via click chemistry, specifically the Huisgen cycloaddition reaction, allowing the biotin-containing tag to attach to 5hmC. The modified 5hmC-containing DNA fragments are then isolated and sequenced. By using this method, the authors observed the gene expression-dependent enrichment of intergenic 5hmC in the mouse cerebellum and the age-dependent acquisition of this modification in specific gene bodies associated with neurodegenerative disorders.

Similarly, Shahal and colleagues used aminooxycyanine 5 (AO-Cy5) as an ARP probe for the fluorescent labeling of 5hmC [68].

##### Modified GLIB Method—hmC-GLIB-IAS Strategy

A chemiluminescent biosensor using 5hmC-specific glycosylation, periodate oxidation, biotinylation and terminal deoxynucleotidyl transferase treatment has been developed [69]. The protocol, named hmC-GLIB-IAS due to its use of isothermal amplification with a poly(C) primer, involves several steps.

In the first step, 5hmC is glycosylated to 5gmC by using βGT. In the second step, 5gmC undergoes selective oxidation with periodate (IO_4_^+^), converting vicinal hydroxyl groups into aldehyde groups, followed by direct chemoselective biotinylation using aldehyde-reactive probes (ARPs), resulting in biotin-5gmC. In the third step, terminal deoxynucleotidyl transferase (TdT)-mediated isothermal amplification generates guanine-rich long DNA products. These guanine-rich DNA products can bind to the hemin cofactor, forming hemin-G-quadruplex nanostructures that produce a chemiluminescent signal in the presence of H_2_O_2_ and luminol. The final step is to measure the chemiluminescence. The hmC-GLIB-IAS strategy enables comprehensive genome-wide analysis with a detection limit for 5hmC of 3.92 × 10^−5^ ng/µL. This method can differentiate 0.1% 5hmC in mixtures of 5hmC-DNA and 5mC-DNA.

##### Multiplex Electrochemical (MEC) Biosensor for 5hmC Detection 

Chen et al. developed a novel multiplex electrochemical (MEC) biosensor for the detection of 5hmC based on the glycosylation of 5hmC and enzymatic signal amplification [70]. First, 5hmC was glycosylated with T4 β-glucosyltransferase (βGT) and subsequently oxidized with sodium periodate. The resulting derivative was then treated with ARP-biotin and incubated with a horseradish peroxidase (HRP)–avidin complex. Electrochemical detection was performed by measuring the oxidation of tetramethylbenzidine.

The MEC biosensor achieved the detection of 5hmC at levels below one nanogram. The authors analyzed the presence of 5hmC in mouse tissue samples and cancer cell lines. The detection limit of the MEC biosensor is 20 times lower than that of commercial kits based on optical measurements.

#### 3.1.4. Glycosylation and Boronic Acid Derivative Modification Followed by PCR Analysis

**Figure 7:**
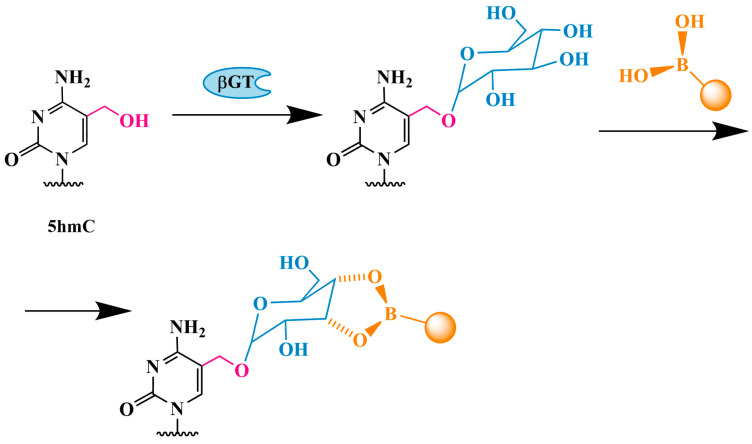
The modification of 5hmC by enzymatic glycosylation and boronic acid for identification by subsequent PCR. The scheme is based on data from [71].

As previously stated, among the currently identified DNA bases, only 5hmC can be specifically converted into 5gmC by βGT. However, the glycosylation of 5hmC in genomic DNA does not inhibit replication by DNA polymerase. Further modification of 5gmC is necessary, and this must be achieved without introducing any DNA damage or byproducts. Boronic acid or its derivatives can selectively bind to the vicinal cis-diol group in 5gmC, increasing its size. This results in a specific stop of DNA polymerase at the modified 5gmC in the DNA matrix. Zhao and colleagues were the first to utilize PCR analysis of boronic acid-modified DNA to detect 5hmC [71]. The authors analyzed the Pax5 gene of genomic DNA from mouse embryonic stem cells that exhibited a high level of 5hmC. The protocol consists of several steps. The initial stage of the process is the glycosylation of 5hmC in double-stranded DNA (dsDNA) by using βGT. In the second step, the modified DNA is treated with boronic acid (Figure 7) or its derivatives, including phenylboronic acid, 3-chlorophenylboronic acid, 2-(2-chlorobenzyl)oxyphenylboronic acid and 3-(dansylamino)phenylboronic acid. The third step is PCR amplification using Taq DNA polymerase. The results yielded by this methodology were corroborated by the deep sequencing of 5hmC. The researchers successfully quantified the distribution of 5hmC in 10 regions in human MRC-5 cells, which exhibit naturally low expression of all three TET proteins. This evidence demonstrates the applicability of this method for the detection of gene-specific 5hmC in biologically relevant samples.

### 3.2. Methods Using Sodium Bisulfite

#### 3.2.1. Conversion of 5hmC into Cytosine-5-Methylene Sulfonate (CMS) (CMS Method)

**Figure 8:**
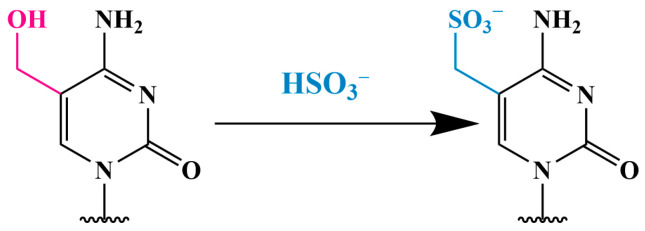
The conversion of 5hmC into cytosine-5-methylene sulfonate (CMS).

To achieve the sensitive and quantitative detection of 5hmC in genomic DNA by using dot blotting, Ko et al. developed the CMS method, which involves the treatment of DNA with sodium bisulfite to convert 5hmC into cytosine-5-methylene sulfonate (CMS) (Figure 8) [72]. The presence of CMS is then detected by using specific antibodies to CMS. Unlike antibodies to 5hmC, which effectively react only with DNA containing high density of 5hmC, antibodies to CMS recognize an average of one 5hmC residue per 201 bp.

After treating genomic DNA with sodium bisulfite, the samples are denatured and blotted onto a nitrocellulose membrane. The membrane is then washed, dried, blocked and incubated with an antibody to 5hmC followed by a secondary antibody conjugated to horseradish peroxidase (HRP). The methylation status of bisulfite-treated DNA is assessed by using the Illumina platform. The methylation status is calculated from the ratio of methylation-specific to demethylation-specific fluorophores by using the BeadStudio Methylation Module. Antibodies to CMS are more sensitive and less dependent on methylation density than antibodies directly to 5hmC, making them suitable for the dot blot analysis of DNA. However, the CMS protocol is relatively lengthy and takes approximately three days to complete [60].

Pastor et al. used a combination of the GLIB method and anti-CMS to identify 5hmC-containing regions in genomic DNA with varying levels of 5hmC [73]. For DNA processed by the GLIB method, they used Helicos single-molecule DNA sequencing, which eliminates the need for amplification and thus avoids systematic errors associated with PCR. For genomic DNA enriched for CMS, they used the Illumina platform because longer read lengths are required to effectively align bisulfite-treated DNA to the genome. The authors observed that both methods yielded similar results—5hmC and 5mC were detected primarily in transcribed regions, especially in exons, which are known to be sites of both high CpG density and high DNA methylation levels.

#### 3.2.2. Oxidative Bisulfite Sequencing Using TET (TAB-Seq) with 5hmC Protection Through Glycosylation

The development of the genomic bisulfite sequencing (BS-seq) protocol for the detection of 5mC residues in single strands of DNA, published by Frommer et al. in 1992, was a major advance in the study of DNA methylation and is currently one of the most widely used methods for the detection of 5mC in epigenetic research [74]. The method combines the bisulfite treatment of genomic DNA under conditions where cytosine (C) is converted into uracil (U), while 5mC remains unreactive, followed by the PCR amplification of the target region within the modified DNA. The resulting PCR products can be sequenced directly, providing an average sequence over many DNA molecules, or cloned and sequenced to provide methylation maps of individual DNA molecules.

**Figure 9:**
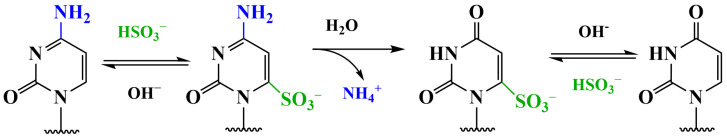
The scheme of bisulfite conversion of cytosine into uracil.

Bisulfite conversion involves a three-step reaction (Figure 9): (1) cytosines are converted into sulfonated cytosines under conditions of low pH and high temperature, (2) sulfonated cytosines are deaminated to sulfonated uracils, and (3) sulfonated uracils are converted into uracils upon alkaline treatment. Subsequent PCR amplifies uracils as thymidine (T). In contrast, 5mC remains unchanged after bisulfite conversion and is amplified as cytosine (C). Since the formation of the DNA double helix protects cytosines from deamination, DNA must first be denatured to a single-stranded state and stripped of proteins to ensure successful modification.

However, traditional bisulfite sequencing cannot distinguish between 5mC and 5hmC because both are resistant to deamination during bisulfite treatment. The bisulfite treatment of 5hmC results in the formation of a stable methyl-sulfonate adduct (Figure 8), which is also read as a cytosine during sequencing [75,76]. Thus, classical bisulfite methylation analysis provides information on the location and quantity of combined 5mC and 5hmC modifications.

**Figure 10:**
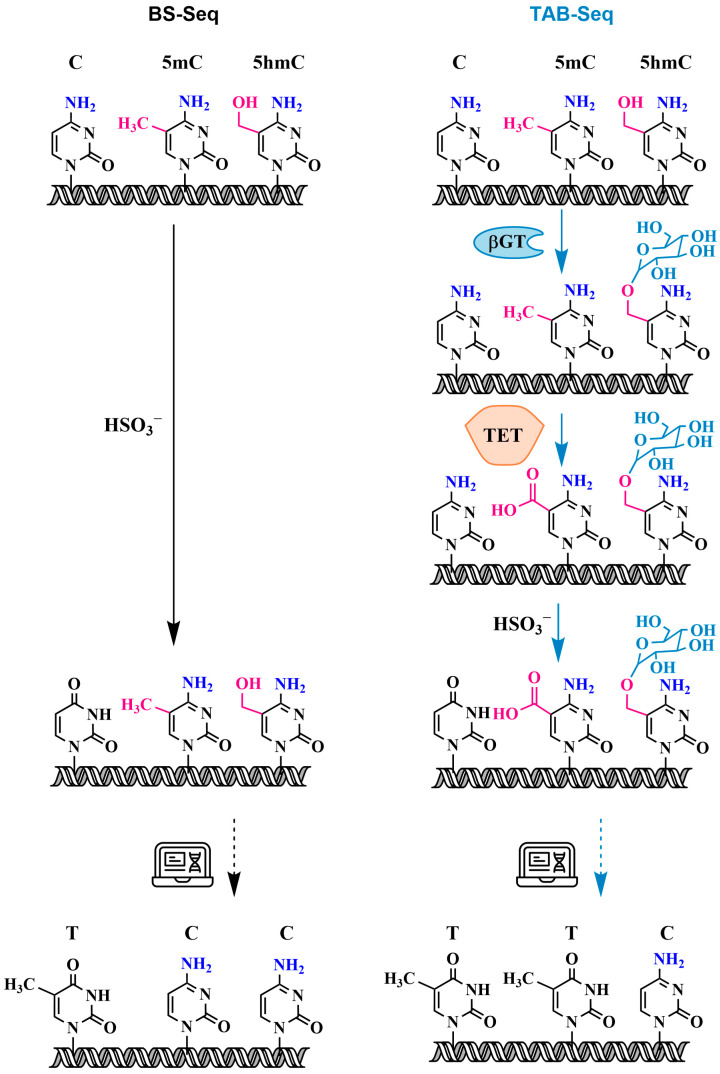
Scheme of bisulfite sequencing method using TET (TAB-seq) versus classical bisulfite sequencing (BS-seq). The scheme is based on the data from [40].

It is known that TET proteins not only oxidize 5mC to 5hmC but also further oxidize 5hmC to 5caC and that 5caC behaves similarly to unmodified cytosine after bisulfite conversion [10]. A new variant of bisulfite sequencing using the TET protein (TAB-seq) was introduced by Yu et al. [40]. This strategy allows for the detection of 5hmC with single-base resolution and is suitable for both genome-wide and locus-specific sequencing. The method is illustrated in Figure 10. The first step of the TAB-seq method is the protection of 5hmC by glycosylation. Following the protection of 5hmC, all 5mC is converted into 5caC during TET oxidation. Subsequent bisulfite treatment converts all cytosines and 5caC derived from 5mC into uracil and 5caU, respectively, leaving only glycosylated 5hmC as the cytosine signal after PCR and sequencing. In combination with the results of traditional bisulfite sequencing, this provides accurate information about the quantity and location within the genome of not only 5hmC but also 5mC.

Nevertheless, despite the selectivity of TAB-seq, methods based on bisulfite sequencing have several limitations. Firstly, bisulfite treatment involves a chemical reaction that degrades a significant portion of DNA due to the need to perform the depyrimidination reaction under acidic conditions at elevated temperatures [77]. This leads to sequencing errors due to context-dependent DNA degradation, which severely limits the utility of bisulfite sequencing when the amount of DNA is small, making it difficult to transition to genome-wide DNA sequencing [78]. Another drawback of TAB-seq is that the incomplete glycosylation of 5hmC or the TET1-mediated oxidation of 5mC can lead to the false-positive detection of 5hmC. Even with high 5mC oxidation efficiency of TET1 (95%), in regions with 80–100% 5mC, only 95% is converted into uracil [79]. Under these conditions, the remaining 5% of unreacted 5mC is falsely identified as 5hmC.

### 3.3. Specific Oxidation of 5hmC to 5-Formylcytosine and Detection of 5fC: Oxidative Bisulfite Sequencing (oxBS-Seq)

**Figure 11:**
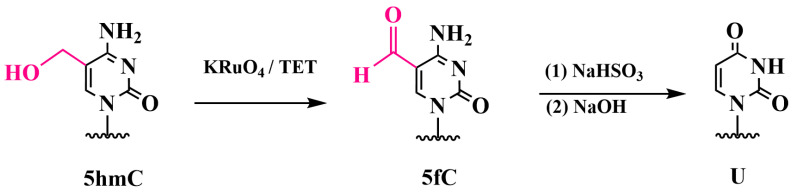
The scheme of 5hmC oxidation to 5-formylcytosine with subsequent bisulfite treatment to obtain uracil. The scheme is based on the data from [80].

Another approach that uses bisulfite conversion to detect 5hmC is oxidative bisulfite sequencing (oxBS-seq). Although this method was developed earlier than TAB-seq, it has been less widely adopted. First introduced by Booth et al. in 2012, oxBS-seq was the first method to quantitatively map 5hmC in genomic DNA with single-nucleotide resolution (Figure 11) [80].

The first step of the oxBS-seq method involves the specific chemical oxidation of 5hmC to 5-formylcytosine (5fC) by using potassium perruthenate (KRuO_4_). Unlike 5hmC, which is resistant to deamination, the bisulfite treatment of 5fC results in deformation and deamination to uracil, which is then read as thymine (T) during sequencing.

Thus, the only base that does not undergo deamination and is therefore read as cytosine (C) after bisulfite treatment is 5mC. The detection of 5hmC is achieved by performing BS-seq (which identifies both 5hmC and 5mC) and comparing the results with those of oxBS-seq (which identifies only 5mC).

Booth and colleagues used oxBS-seq to map and quantify 5hmC at CGIs in mouse embryonic stem cells, identifying 800 5hmC-containing CGIs with an average hydroxymethylation level of 3.3%. One year later, the same research group proposed an optimized second-generation protocol that can be completed in two days, including sample preparation [76]. The oxBS-seq method can be combined with several analytical tools successfully used for BS-seq: various DNA sequencing methods (Sanger sequencing, pyrosequencing and high-throughput sequencing) and whole-genome sequencing techniques. When used in conjunction with next-generation sequencing, this method is platform-independent and compatible with the single-base resolution analysis of both 5mC and 5hmC, applicable to both whole-genome and targeted-region formats.

Another example of using oxidation for 5hmC detection is the rolling circle amplification (RCA) fluorescence method. This method is based on the oxidation of 5hmC to 5fC, followed by the conversion of 5fC into uracil (U) in conjunction with ligation-mediated RCA [81]. This approach demonstrates high sensitivity, with a detection limit as low as 34.8 fM. It can differentiate a 5hmC level as low as 0.01% in a mixture and can be used for serum-based analysis.

#### Chemiluminescent Method for Detecting 5fC in DNA

**Figure 12:**
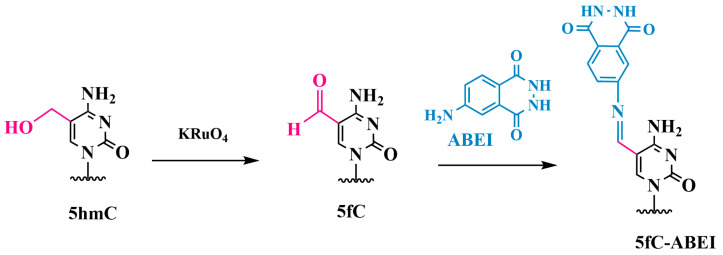
The scheme of 5hmC oxidation to 5-formylcytosine with subsequent electrochemiluminescent labeling with N-(4-aminobutyl)-N-ethylisoluminol (ABEI). The scheme is based on the data from [82].

Ma and colleagues developed a quantitative method for detecting 5hmC in DNA, based on selective electrochemiluminescent labeling (ECL) with the prior oxidation of 5hmC to 5fC by using KRuO_4_ [82]. The main steps of the method include a thiolated probe (a 35-mer oligonucleotide) to capture 5hmC-containing target DNA, which self-assembles on a gold surface. The 5hmC in the target DNA is selectively converted into 5fC by oxidation with KRuO_4_ and then processed with N-(4-aminobutyl)-N-ethylisoluminol (ABEI) (Figure 12). The aldehyde group of 5fC reacts with the amine group of ABEI to form a Schiff base. The ABEI-labeled target DNA hybridizes with the capture probe on the electrode, resulting in ECL emission. The method achieved an exceptionally low detection limit of 1.4 × 10^−13^ M for 5hmC in DNA. The ECL method has been demonstrated to be useful for the quantitative detection of 5hmC in serum samples.

### 3.4. Electrochemical Magnetobiosensor for Analyzing the Presence of 5hmC by Using Amplification

**Figure 13:**
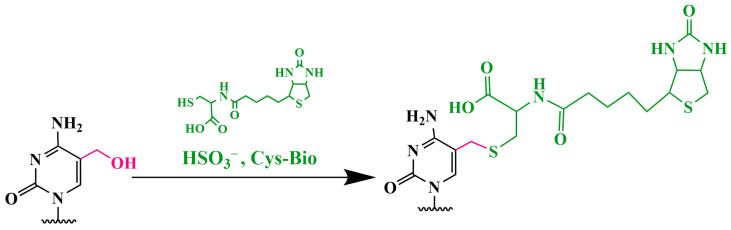
The scheme of 5hmC bisulfite-mediated biotinylation. The scheme is based on the data from [83].

The electrochemical magnetobiosensor, developed in 2019, demonstrates high sensitivity, with a detection limit for 5hmC of only 9.06 fM [83]. The analysis involves two sequential steps: (1) the one-step bisulfite-mediated biotinylation of 5hmC in DNA (Figure 13); (2) a dual amplification strategy for biotinylated 5hmC DNA involving TdT-catalyzed polymerization and subsequent amplification mediated by the redox recycling of Ru(III).

In this electrocatalytic reporter system, Ru(NH₃)_6_^3^⁺ acts as the primary electron acceptor, while Fe(CN)_6_^3−^ serves as the secondary electron acceptor. Ru(NH_3_)_6_^3+^ interacts electrostatically with the negatively charged phosphate backbone of DNA on the surface of the magnetobiosensor and is immobilized on a screen-printed carbon electrode (SPCE) by using a magnetic field. Ru(III) is electrochemically reduced to Ru(II). Excess Fe(CN)_6_^3−^ enhances the current flowing to Ru(NH_3_)_6_^3+^ by oxidizing Ru(II), resulting in the recycling of Ru(III) and the generation of an enhanced electrocatalytic current. This biosensor can distinguish 5hmC from C and 5mC and successfully detects 5hmC in living cells.

### 3.5. Oxidation of 5mC and 5hmC to 5-Carboxycytosine (5caC)—Pyridine Borane Sequencing with TET (TAPS) and KRuO₄ (CAPS)

**Figure 14:**
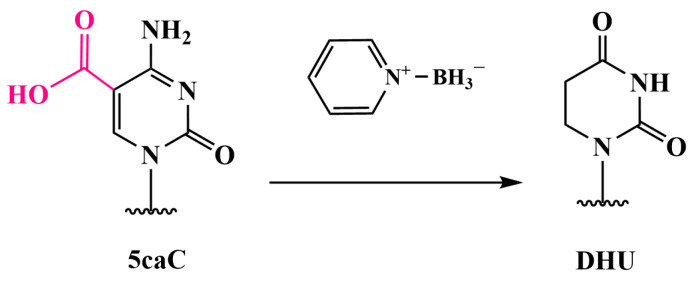
The scheme of 5caC reduction to dihydroxyuracil (DHU) with pyridine borane. The scheme is based on the data from [84].

In 2019, Liu et al. introduced a method for detecting 5mC and 5hmC with single-base resolution, known as TET-assisted pyridine borane sequencing (TAPS), which avoids the use of sodium bisulfite [84]. This method represents a breakthrough in methylation detection by overcoming the limitations of bisulfite conversion. The TAPS protocol involves the oxidation of 5mC and 5hmC to 5caC with TET, followed by the reduction of 5caC to dihydroxyuracil (DHU) with pyridine borane (Figure 14). Subsequent PCR amplification converts DHU into thymine, resulting in a C-to-T transition for 5mC and 5hmC. Technically, TAPS also detects two minor DNA modifications, 5fC and 5caC, although these are present at extremely low levels in mammalian genomes (less than 0.002% of the total cytosine) [85].

**Figure 15:**
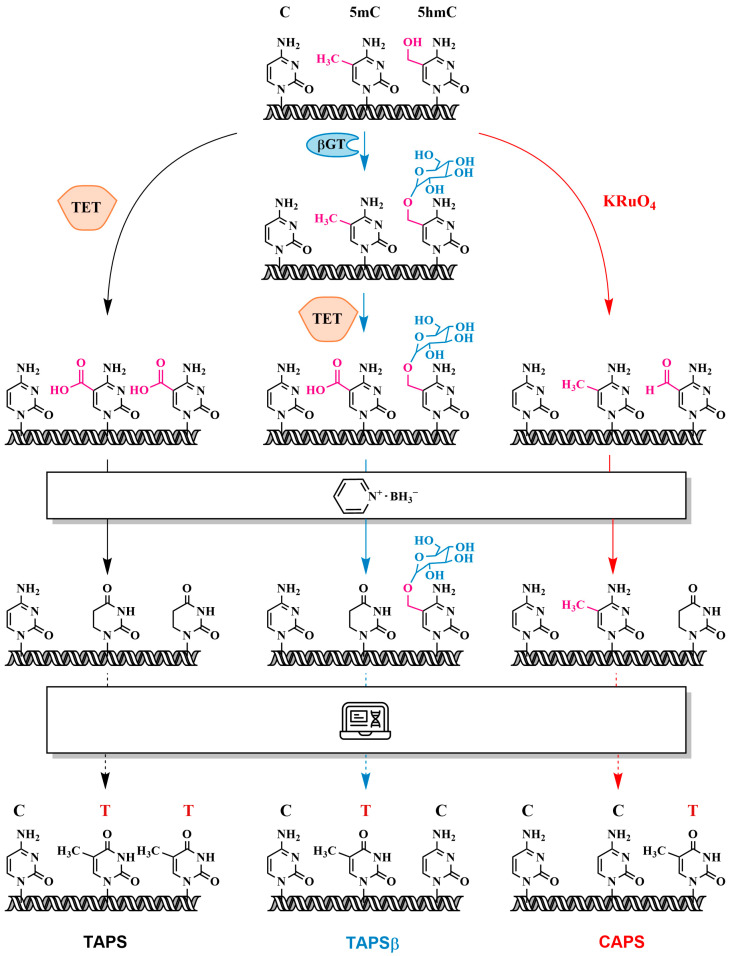
Comparison of bisulfite sequencing and related methods with TAPS and CAPS for sequencing DNA containing 5mC and 5hmC. Figure based on data from [84].

Alternatively, potassium ruthenate (KRuO_4_) can be used to specifically oxidize 5hmC to 5fC. The chemical oxidant KRuO_4_, used in the oxBS-seq method, was utilized in an approach known as chemical pyridine borane sequencing (CAPS). Compared with bisulfite sequencing, TAPS shows improved mapping rates, more uniform coverage, and reduced sequencing costs (see Figure 15). Unlike the harsh bisulfite treatment, TAPS preserves long DNA molecules of more than 10,000 bp, which is critical for long-read sequencing [85]. TAPS is compatible with a variety of subsequent analyses, including pyrosequencing, PCR, methylation-sensitive restriction digestion, MALDI mass spectrometry, microarray analysis and whole-genome sequencing. For example, TAPS has been used for the whole-genome mapping of 5mC and 5hmC in mouse embryonic stem cells [85].

Building on the TAPS method, Cheng et al. introduced TAPS with endonuclease enrichment (eeTAPS) in 2021 [86]. This approach employs an endonuclease that cleaves DNA at DHU sites to hydrolyze DNA modified by TAPS, providing more comprehensive coverage of methylated CpG (mCpG) sites. eeTAPS enables the accurate detection of 87% of mCpG sites in the mouse genome, with a sequencing depth equivalent to four times that of whole-genome sequencing. Because TAPS can maintain long DNA without breaks, it could be extremely valuable when combined with long-read sequencing technologies such as SMRT sequencing and nanopore sequencing. In 2022, Chen et al. integrated TAPS with PacBio single-molecule real-time sequencing to develop whole-genome long-read TAPS (wglrTAPS) [87]. In addition, Siejka-Zielińska et al. optimized TAPS for cfDNA (cfTAPS) in 2021 [88].

### 3.6. Analysis of 5hmC Presence by Chemical Oxidation with Peroxotungstate to Trihydroxythymine (thT)

**Figure 16:**
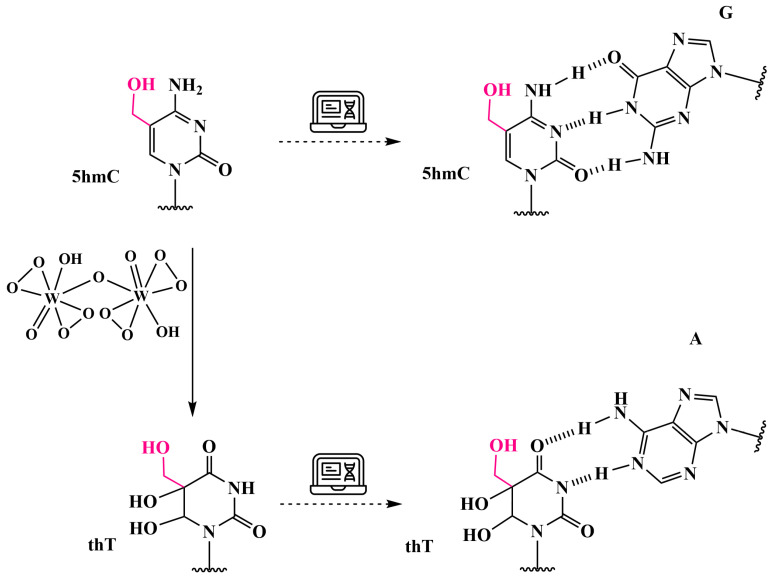
Scheme of 5hmC analysis by peroxotungstate oxidation to trihydroxylate-thymine (thT) followed by sequencing (seq). Figure based on data from [90].

In 2011, Okamoto et al. demonstrated that peroxotungstate oxidation is an effective method for the detection of 5hmC in DNA, distinguishing it from its epigenetic predecessors, 5mC and unmethylated C [89]. The C5-C6 carbon atoms of the allylic alcohol fragment in 5hmC are oxidized by peroxotungstate to produce an epoxide, which upon subsequent nucleophilic attack by water produces a trihydroxylated and 4-deaminated product, trihydroxythymine (thT) (Figure 16) [90]. The oxidized DNA strand is readily fragmented into thT nucleotides when treated with hot piperidine, allowing for the identification of single-stranded DNA containing 5hmC as a hydrolysis band on acrylamide gel electrophoresis after peroxotungstate and piperidine treatment.

It was found that thT is tolerant to the incorporation of dATP as a substrate during elongation [90]. To selectively discriminate thT from C and 5mC, ligation was proposed, which can detect single-nucleotide differences [91]. In addition, peroxotungstate can be used for the selective and direct conversion of 5hmC into thT, bypassing intermediates such as 5fC [91]. However, a major problem is non-specific amplification, especially when using PCR-based methods, which limits both specificity and sensitivity.

The two-phase amplification system proposed in the POM-TPAS method combines single-molecule and two-phase PCR amplification. The underlying principle of this method is as follows: Two double-stranded DNAs (dsDNAs) containing C, 5mC and 5hmC at the same position are identified as dsDNA-C, dsDNA-5mC and dsDNA-5hmC, respectively. In this context, dsDNA-X represents either dsDNA-C or dsDNA-5mC. The 5hmC on dsDNA-5hmC is specifically oxidized by potassium peroxotungstate to the trigydroxylated and 4-deaminated product thT, while dsDNA-X remains unchanged. Therefore, differences between thT and C/5mC can be detected by ligation reactions.

However, it should be noted that this oxidation strategy cannot differentiate between 5hmC and T due to the similar base-pairing ability of thT and T. Disadvantages of the method include non-specific amplification and false positives, as well as complex primer design and the need for labeled nucleic acid probes.

To selectively distinguish thT from C and 5mC, the oxidation of 5hmC with peroxotungstate followed by loop-mediated isothermal amplification based on ligation can be employed [92]. In 2022, Zhang et al. developed a mismatched base ligation strategy in which a base mismatching with the target sequence is introduced at the third position from the 3′ end of probe B [93]. The mismatched base interacts with the ligase, providing high specificity to the ligation process.

## 4. Methods Based on the Use of DNA-Modifying Enzymes

### 4.1. Use of Restriction Endonucleases

Historically, the first direct method for detecting methylated cytosine in DNA was introduced in 1979 [94]. The method involved the use of a pair of site-specific restriction endonucleases (REs), one of which is methylation-sensitive (MSRE) and the other is an isoschizomer of the first, recognizing the same site but hydrolyzing DNA regardless of the methylation status of the recognition site. The pair of enzymes proposed in the study [94] were HpaII (MSRE) and MspI (the insensitive isoschizomer), which recognize and hydrolyze the 5′-C↓CGG-3′ site. This remains the most commonly utilized pair of REs for the analysis of DNA methylation. The hydrolysis of the same DNA sample with the REs HpaII and MspI followed by electrophoretic analysis of the cleavage products reveals the distribution of methylation sites in the DNA sample.

Further RE-based systems were introduced with glucosyl-5-hydroxymethylcytosine-sensitive restriction endonucleases. In 2011, Kinney et al. discovered that MspI cleaves 5hmC-containing CCGG sites but does not digest 5gmC-containing DNA [95]. The protocol starts with 5hmC glycosylation, subsequently employing a glucosyl-5-hydroxymethylcytosine-sensitive RE (MspI) that is capable of digesting DNA when C, 5mC or 5hmC is present within its recognition site in a CpG context (yet this is impeded by the presence of 5gmC) to generate two distinct sample populations. The isoschizomer of the first RE (HpaII) is blocked by all possible modifications (5mC, 5hmC and 5gmC) in a CpG context, and therefore only hydrolyses a DNA sequence where an unmodified cytosine is present. Furthermore, more complex protocols utilizing a combination of glycosylation and RE have been developed, with DARESOME being a notable example [96]. These results confirmed that the digestion of glycosylated DNA can be used to determine the tissue-specific presence of 5hmC and 5mC in mammalian genomic DNA. After hydrolysis with the combination of REs (MspI/HpaII), any suitable method for the analysis of DNA fragments can be used, typically PCR amplification with primers flanking the restriction site. If the DNA is methylated, cleavage will not occur, resulting in successful PCR amplification. Modern approaches use, e.g., real-time PCR with the intercalating dye SYBR Green and primers flanking the restriction site [97].

The detection of 5hmC using restriction endonucleases is only applicable to certain sequences. AbaSI, a methylation-sensitive DNA RE, can cleave DNA at a distance of 11–13 bp from βGT-glycosylated 5hmC when another cytosine is present at a distance of 9–11 bp in the opposite direction [98]. The AbaSI-based method allows for the detection of 5hmC with relative quantification and single-base resolution in less than 40% of cases.

The method using methylation-sensitive REs is often applied to the analysis of CGIs. In 2007, Shen et al. conducted the DNA methylation profiling of promoter regions, identifying the methylation status of 6177 genes by using restriction enzyme-based CGI amplification and microarrays [6]. The advantages of using restriction endonucleases over other methods for detecting 5mC in DNA samples include high specificity, mild reaction conditions, the ability to analyze small amounts of DNA and simple primer design. No special equipment other than a real-time PCR system is required. Disadvantages include the limitation to analyze DNA containing specific sites limited by the recognition sites of REs. In addition, incomplete hydrolysis can lead to false-positive results [99].

Zhang et al. combined the use of MSREs, oxidation of 5hmC to 5fC and bisulfite conversion in their method for the quantitative detection of site-specific 5hmC in genomic DNA samples [100]. The DNA was first treated with HpaII, which leaves 5mC and 5hmC intact. The HpaII-cleaved fragments were then oxidized with KRuO_4_ and subjected to bisulfite treatment. As a result, 5hmC was selectively oxidized to 5fC, and both C and 5fC residues were converted into U, while 5mC remained unchanged. Therefore, the difference in epigenetic modification between 5mC and 5hmC can be effectively converted into a difference between 5mC and U. The subsequent ligation-based PCR offers high selectivity and allows for the detection of single-base differences in DNA molecules.

### 4.2. Use of DNMT1 Methyltransferase: EnIGMA Method

The EnIGMA (Enzyme-assisted Identification of Genome Modification Assay) method developed by Kawasaki and colleagues allows for the simultaneous identification of 5mC and 5hmC by using DNMT1 [101]. The DNMT1 methyltransferase methylates cytosines in hemimethylated CpG sites, but it does not methylate hemi-hydroxymethylated CpG sites or unmethylated CpG sites.

Genomic DNA is first digested with a specific restriction enzyme. The hydrolyzed DNA is then end-repaired and ligated with adapter DNA in the form of a dU-containing hairpin structure, followed by cleavage with the USER enzyme (uracil DNA glycosylase and endonuclease VIII). In parallel, the hydrolyzed DNA is dephosphorylated and directly ligated with adapter DNA in the form of a hairpin structure. The DNA is then treated with a DNA polymerase to synthesize the complementary strand and subjected to DNMT1 treatment, followed by bisulfite conversion and PCR. The resulting PCR product is subjected to sequencing, and the corresponding CpG sites are compared to determine whether the cytosines in the original DNA were methylated, hydroxymethylated or unmodified.

### 4.3. Use of DNA Deaminases: AMD-Seq and ACE-Seq Methods

Methods using other DNA-modifying enzymes, specifically DNA deaminases, have been developed. The AID/APOBEC family of proteins are DNA deaminases that catalyze the deamination of cytosine to uracil in single-stranded DNA (see Figure 1). Several members of this family have been shown to discriminate between different states of cytosine modification, such as wild-type APOBEC3A (wtA3A) [102]. It was also found that wtA3A deaminates 5hmC to a lesser extent and has no deamination activity towards glycosylated 5hmC. The resulting products can then be analyzed by high-throughput sequencing on Illumina platforms following library preparation. Based on the properties of wtA3A, deamination-based sequencing methods were developed, i.e., AMD-seq and ACE-seq, to map 5hmC in DNA with single-base resolution. However, these methods require prior treatment of DNA with βGT to convert 5hmC into 5gmC. In one study, a protein called eA3A-v10 was constructed by replacing amino acid residues in key loops 1 and 7 of wtA3A, which are involved in positioning cytosine through direct interaction with the pyrimidine ring [103]. The mutant enzyme eA3A-v10 demonstrated effective deamidation activity towards C and 5mC but no deamidation activity towards 5hmC in various DNA sequences. Based on the properties of eA3A-v10, the authors developed a sequencing method designated as Single-Step Deamination Sequencing (SSD-seq) [103].

## 5. Affinity-Based Methods: Immunostaining and Immunoprecipitation

Immunostaining is a widely employed technique in cell biology to detect proteins and metabolites in situ in fixed cells. This method relies on the specificity of antibodies to recognize and bind to a selected target and involves immunolabeling, followed by visualization. Antibodies have been developed to recognize the modified cytosine 5mC and its derivatives, 5hmC, 5fC and 5caC [104]. To enhance detection, signals from primary antibodies can be amplified by using secondary antibodies conjugated to fluorophores for immunofluorescence or to other molecules for chemiluminescence. Immunostaining can provide information regarding the spatial distribution and level of DNA methylation within the nucleus. Although the resolution is still relatively low from a genomic perspective, modern microscopy and imaging analysis techniques can provide sufficiently detailed spatial information from immunostained areas. Immunostaining does not allow for the precise identification of loci containing 5hmC, but not all 5hmC sites require precise quantitative assessment for disease analysis. Immunostaining complements genomic approaches that allow for the assessment of DNA methylation at specific sequences; however, it is unable to provide a global nuclear spatial context. Immunoprecipitation and immunostaining methods can be hampered by antibody cross-reactivity, potentially leading to non-specific binding and inaccurate detection. In addition, they provide semi-quantitative results rather than absolute quantification, making it difficult to measure the exact levels of 5hmC in the genome.

The enzyme-linked immunosorbent assay (ELISA) protocol for the detection of 5hmC is a well-established and convenient methodology. A number of commercially available ELISA kits enable the detection and quantification of 5hmC in genomic or in vitro modified DNA. The method is based on the adsorption of DNA onto a solid surface and the subsequent detection of 5hmC by using specific antibodies. The accuracy of the results is contingent upon the quality of the 5hmC antibody, the conjugated secondary antibody, and successful washing. While this method is suitable for the global detection and quantification of 5hmC in the analyzed DNA sample, including whole-genome methylation profiling, it is not possible to ascertain the location of the modification. Nevertheless, this method can be employed for preliminary high-throughput screening and approximate estimation of total DNA hydroxymethylation. The detection limit of these assays is typically higher than 0.02% 5hmC/dNTPs (in 100 ng of DNA), which constrains their applicability in the analysis of human cancer tissues. Furthermore, these assays only yield relative 5hmC levels; for absolute measurements, a calibration curve is needed [105].

Immunofluorescence analysis provides a visual representation of the content and distribution of 5hmC-containing DNA within individual cells. However, nuclear DNA is typically wrapped around nucleosomes, packaged into chromatin, and further associated with many functional proteins. This creates barriers to antibodies that are capable of selectively recognizing 5hmC in DNA. Zhong et al. developed a strategy to assess the accessibility of 5hmC-containing DNA in chromatin in situ by “unmasking” the DNA by treatment with HCl to release the DNA from bound proteins [106]. It is also important to note that despite the availability of commercially available antibodies that specifically recognize 5hmC, these antibodies bind preferentially to highly 5-hydroxymethylated sites rather than to single 5hmC sites.

Single-molecule immunofluorescence visualization allows for the detection of 5hmC in genomic DNA with high sensitivity and provides rapid results. Du et al. proposed hybridization-based single-molecule immunofluorescence visualization for the detection of 5hmC in DNA [107]. By using a DNA probe to capture the target DNA fragment and antibodies specific for 5hmC to detect the 5hmC modification in DNA, the authors were able to detect fluorescent signals from secondary antibodies at the single-molecule level.

To detect the presence of 5hmC in DNA from human frontal cortex tissue, Jin and colleagues employed an antibody against 5hmC for dot blotting and in immunoprecipitation experiments with prior DNA denaturation to enhance the efficiency of immunoprecipitation [108]. 5hmC is known to be abundant in gene promoters and bodies but is generally absent from non-genic regions of DNA. Antibody-based methods for detecting 5hmC do not provide sequence information on the hydroxymethylated regions of DNA, but this can be achieved with the (h)MeDIP-seq sequencing of hydroxymethylated DNA after immunoprecipitation, in which the hydroxymethylated fraction of genomic DNA is captured by using an antibody against 5hmC. After hydroxymethylated DNA fragments are bound to a specific antibody, they are isolated, purified, amplified, sequenced, and mapped to the reference genome by using whole-genome sequencing. When searching for differentially hydroxymethylated loci, these methods are used as a preliminary step to “pull out” the hydroxymethylated DNA fraction from the sample, thereby enriching the fraction of target DNA. Therefore, this method is referred to as an “affinity enrichment method”. It is important to note that (h)MeDIP-seq profiles, like chromatin immunoprecipitation (ChIP)-seq profiles, only indicate the relative distribution of the modification within the sample and therefore cannot be used to infer absolute quantitative differences between samples or antibodies [109].

By using (h)MeDIP-seq, Stroud and colleagues generated a genome-wide map of 5hmC in human embryonic stem cells by the immunoprecipitation of hydroxymethylated DNA regions followed by massive parallel sequencing [28]. The DNA fragments ligated to Illumina adapters were used for immunoprecipitation, and it was demonstrated that 5 μg of DNA was sufficient for immunoprecipitation. The 5hmC-enriched DNA was sequenced by using the Illumina platform. By using (h)MeDIP-seq for genome-wide analysis of 5hmC and 5mC in differentiated cell types, Mellén and colleagues identified methyl-CpG-binding protein 2 (MeCP2) as the primary 5hmC-binding protein in the brain and showed that MeCP2 binds with equal affinity to both 5hmC- and 5mC-containing DNA [110]. The R133C mutation in MeCP2, which causes Rett syndrome, predominantly inhibits 5hmC binding. In this case, 0.5–1 μg of DNA was sufficient for (h)MeDIP-seq, and sequencing was also performed on the Illumina platform.

## 6. Next-Generation Sequencing

High-throughput parallel sequencing on next-generation sequencing (NGS) platforms has replaced Sanger sequencing and is actively employed to analyze 5hmC after enzymatic or chemical modification for selectivity and enrichment steps. This is reflected in 5hmC detection methods such as MBD-seq [60,61], TAB-seq [40,74], GLIB [66], oxBS-seq [80], TAPS [84], CAPS [85], eeTAPS [86], AMD-seq, ACE-seq [102], JBP1-seq [65], SSD-seq [103], and (h)MeDIP-seq [28], which have been discussed previously. All of these methods produce a large number of data, but the interpretable signal comes from arrays of molecules.

The move to the latest (or third-generation) sequencing technology, which performs single-molecule DNA sequencing, allows for the analysis of long single sequences. Third-generation genome sequencing (TGS) methods, also known as long-read sequencing methods due to their ability to continuously analyze long DNA molecules, include single-molecule real-time (SMRT) sequencing on the PacBio platform and nanopore sequencing using Oxford Nanopore Technologies (ONT).

An illustrative example of combining the TAPS method with SMRT sequencing is the wglrTAPS approach [87]. In addition, TET-assisted pyridine borane sequencing with TGS analysis has been designated lrTAPS (used with both SMRT and ONT) [85]. Furthermore, TGS enables the direct detection of modified DNA bases at specific genomic locations without chemical modification and signal averaging by providing additional analysis of the primary signal. This capability will be discussed in further detail below.

### 6.1. Single-Molecule Real-Time (SMRT) DNA Sequencing

The most optimal approach would be the direct detection of modified bases. This has been achieved with SMRT sequencing, which permits the detection of methylated bases without bisulfite conversion, including 5mC and 5hmC, as well as 6-methyladenine, 4-methylcytosine, 8-oxoguanine and others [111]. In SMRT sequencing, the DNA molecules are prepared by circularizing them, whereby a closed ring of native double-stranded DNA is created by ligating hairpin adapters to the ends. During SMRT sequencing, the DNA polymerase repeatedly uses the circular DNA template. The exact number of cycles required to reliably identify a modified base depends on the fragment size and the characteristics of the polymerase, which catalyzes the incorporation of fluorescently labeled nucleotides into the complementary strand, including those with modified bases present in the template strand [112]. The alteration in the conformation sets of the modified heterocyclic base leads to variations in the time required to form a complementary pair with the incoming nucleotide, which in turn affects the rate of incorporation of the new base into the growing DNA strand. The detection of DNA polymerase is accomplished by measuring the time of fluorescence emission and the duration of the fluorescence pulses, thereby providing information on the kinetics of polymerase activity. The incorporation of a specific nucleotide is detected by a pulse of fluorescence whose wavelength identifies the nucleotide. The pulse ends when the fluorophore attached to the terminal phosphate of the nucleotide is cleaved by the polymerase before it translocates to the next template base in the DNA. The typical synthesis rate during SMRT sequencing is one to three bases per second. The sequencer monitors not only the fluorescence pulses associated with each incorporated nucleotide but also the time interval between events, known as the inter-pulse duration (IPD). Variations in IPD reflect kinetic changes in enzyme activity and correlate well with the occurrence of modifications in the DNA [113,114].

### 6.2. Nanopore Sequencing (ONT)

Nanopore sequencing, developed by Oxford Nanopore Technologies Ltd. as a fourth-generation DNA sequencing technology, is a powerful method for the rapid analysis of long sequences without the need for PCR amplification or the chemical labeling of the sample. ONT sequencing differs from all previous methods described in this review, in that nucleotides are detected directly as they pass through a protein nanopore (a pore with a diameter of 10^−9^ m, comparable to the size of a single strand of nucleic acid) stabilized in an electrically resistant membrane. A voltage is applied to the membrane, and sensors record changes in ionic current caused by nucleotides occupying the pore in real time as the DNA molecule passes through [115].

A major drawback of ONT sequencing, as with other techniques for sequencing long strands of DNA, is the relatively high error rate, which ranges from 5% to 20% [116]. However, success has been achieved in detecting and mapping 5mC and 5hmC on individual DNA strands by using nanopore-based real-time sequencing technology [117]. The rapid advances and analysis of pore protein modifications in ONT suggest that accuracy will continue to improve [118]. Enhanced overall accuracy will increase the reliability of the direct detection of modified bases and the efficacy of deep learning modification detection algorithms [119]. The current analysis demonstrates a high correlation with bisulfite sequencing for 5mC detection, requires less read depth and achieves enhanced reliability in biologically significant regions of the genome, such as CpG-rich promoters.

### 6.3. Whole-Genome Sequencing

It is important to separately discuss whole-genome analysis for the presence of the 5hmC modification, which requires a larger amount of sample. Han et al. proposed a 5hmC sequencing method that allows for 5hmC profiling across the genome based on selective chemical labeling, similar to the GLIB method, but using a limited amount of genomic DNA, for which about 1000 cells are sufficient (this method is called nanoq-hmC-Seal) [120]. The sequencing adapters are integrated by transposase-catalyzed DNA fragmentation [121]. The enzyme βGT is then used to transfer a synthesized glucose fragment containing an azide group from UDP-6-N3-Glc to 5hmC in duplex DNA, thereby forming β-6-azido-glucosyl-5-hydroxymethylcytosine (N3-5gmC). Subsequently, the azide group is chemically modified with biotin, enabling the effective capture of biotin-labeled 5hmC-containing DNA fragments from a pool of random DNA fragments by using avidin beads. This affinity enrichment and the subsequent PCR amplification creates a library that is then subjected to the high-throughput sequencing of 5hmC-containing DNA fragments. In another study, the authors quantitatively determined 5hmC in eight types of mouse spermatogenic cells by using HPLC-MS/MS and then used the chemical labeling method UDP-6-N3-Glc with βGT followed by biotin labeling and enrichment combined with deep sequencing [122]. The resulting data mapped the distribution of 5hmC in different genomic regions. It was found that 5hmC is differentially mapped and dynamically changes in genomic regions associated with the regulation of gene expression, piRNA precursor genes and repetitive elements.

An interesting approach was recently proposed by Bai et al. [123]. This method, designated as SIMPLE-seq, is a scalable technique for the simultaneous analysis of 5mC and 5hmC from thousands of individual cells. Based on the orthogonal labeling and registration of mutation signals “C-to-T” from 5mC and 5hmC sites, SIMPLE-seq detects these two modifications in the same molecules in single cells, allowing for the objective analysis of DNA methylation dynamics in heterogeneous biological samples. The authors applied this method to mouse embryonic stem cells, human peripheral blood mononuclear cells and mouse brain in order to obtain common epigenomic maps at single-cell and single-molecule resolution.

## 7. New, Original Methods for Quantitative Assessment of 5hmC

New and original methods for the quantitative assessment of 5hmC have been developed by using DNA nanotechnology, which allows for the visualization of structural changes at the nanoscale. Optical mapping methods involve the selective labeling of detectable DNA modifications with fluorescent reporter molecules and can provide epigenetic information about individual DNA molecules up to 1 million bp in length [31]. By stretching DNA molecules into a linear configuration, detectable DNA modifications can be directly visualized by using high-resolution fluorescence microscopy. Gabriel et al. developed an optical mapping method for “long-read” single molecules, which generates hybrid genetic/epigenetic profiles of native chromosomal DNA [124]. Fluorescence microscopy enables the simultaneous detection of genetic and epigenetic information on the same molecule, with each feature being labeled with a distinct color. A genetic barcode is created by the enzymatic labeling of a DNA sequence at a specific motif, while an additional layer of epigenetic information is created by modifying 5hmC through a specific chemical–enzymatic reaction. To obtain a whole-genome profile of epigenetic modifications, the genomic positions of 5hmC tags are mapped, and the genetic tags are compared to a reference sequence. This methodology has been integrated into the genome mapping technology commercialized by BioNano Genomics Inc. (San Diego, CA, USA), which is based on the expansion of fluorescently labeled DNA molecules in arrays of nanopores.

In a separate study, a regulated DNAzyme motor was developed for the quantitative assessment of 5hmC triggered by strand displacement amplification (SDA) [125]. The sites containing 5hmC as the primary target were used as a matrix during DNA synthesis and specifically labeled with DNA primers. These matrices were amplified into a large amount of single-stranded DNA (the secondary target) via the SDA reaction, which activated the DNAzyme motor. As the quantity of secondary target DNA increased, the DNAzyme motor gradually regained its activity, enabling the continuous cleavage of strands of auxiliary nucleic acid labeled with quenching probes, resulting in the restoration of the electrochemiluminescence signal. Calculations made by the authors indicate that the detection limit (limit of detection, LOD) of this method for 5hmC is 0.49 fM. This suggests that 5hmC can be detected at 0.1% content in a 0.17 ng DNA sample or at 0.01% content in a 1.7 ng DNA sample. This technique exhibits an exceptionally low detection limit, although it is challenging to perform.

The two epigenetic marks 5mC and 5hmC together provide a more accurate prognosis for cancer recurrence and survival than when assessed separately. However, distinguishing and quantitatively assessing these two methylation variants is challenging due to their similar structures. To identify and quantify 5mC and 5hmC in random DNA sequences, a nanoconfigured ECL biosensor based on CPDs@SiO_2_ was developed [126]. The hydroxymethyl group of 5hmC was modified with UDP-6-N3-Glc by using β-GT. Subsequently, specific double-stranded DNA (dsDNA) was attached to each modified residue by click chemistry. Recombinase polymerase amplification (RPA) was performed by using primers specific for the inserted dsDNA fragments, followed by transcription with T7 RNA polymerase. This process generated RNA fragments that activated the CRISPR/Cas13a system, which then triggered electrochemiluminescence by hydrolyzing the fluorophore-labeled DNA immobilized on the biosensor. To detect 5mC, it was converted into 5hmC by using TET1. The authors were able to achieve selective detection of 5hmC.

## 8. 5hmC as a Biomarker for Diagnosing Cancer and Neurodegenerative Diseases

For decades, 5mC has been considered the “fifth base” of the genome, but now, 5hmC is recognized as the “sixth base” in mammalian genomes, in addition to adenine, cytosine, thymine, guanine, and 5mC. Recent research has demonstrated that 5hmC is an important epigenetic marker involved in oncogenesis, embryogenesis, central nervous system function and various pathological conditions [31,32].

The measurement of 5hmC levels has the potential to become a valuable tool for the early diagnosis of cancer. Malignant tumors are known to lose many features of normal tissue/cellular architecture and become dedifferentiated. If 5hmC is considered a marker for cell- or tissue-specific genes, its loss in tumor cells would be a logical event. The question is whether the loss of 5hmC in gene bodies is a cause or a consequence of malignant transformation and whether other factors contribute to its loss. To provide evidence that reduced 5hmC levels are a cause and not just a consequence of malignancy, Koh and colleagues introduced normal and TET-deficient stem cells into mice [127]. While normal stem cells formed highly differentiated benign teratomas, TET-deficient stem cells developed highly aggressive tumors that exhibited a high proliferative state.

Low levels of 5hmC have been observed in a number of solid tumors, including aggressive forms of lung, kidney and colon cancers, as well as melanoma, brain tumors and hepatocellular carcinoma [128,129,130,131,132,133]. In squamous cell lung cancer, 5hmC levels were found to be reduced 5-fold, while in brain tumors, the reduction was 30-fold compared with normal tissue [134]. It is important to note that in some types of malignancies, there is not only a significant loss of 5hmC, but also disease-specific changes in the level and distribution of 5hmC not only in genomic but also in extracellular DNA (ecDNA). Song and colleagues observed that lung cancer leads to a progressive global loss of 5hmC in ecDNA, whereas hepatocellular carcinoma and pancreatic cancer lead to disease-specific changes in the distribution of 5hmC in ecDNA [135]. The authors employed the method described in [67], which utilizes βGT to selectively label 5hmC with a biotin via an azide-modified glucose for the isolation of 5hmC-containing DNA fragments for subsequent sequencing.

It is important to note that the determination of 5hmC in DNA is not applicable for disease diagnosis in certain types of cancers, including breast cancer, primary diffuse large B-cell lymphoma of the testis, melanoma and prostate cancer [136,137,138,139]. For example, a 5hmC-based biomarker compared with blood prostate-specific antigen (PSA) levels was less effective in detecting prostate cancer than using a PSA threshold of 2.5 ng/mL. However, 5hmC can identify the characteristics of aggressive subtypes of prostate cancer that could serve as disease biomarkers [140]. The preparation of sequencing libraries and the enrichment of 5hmC were conducted in accordance with the previously described methodology [135]. For many other cancers, the location and density of 5hmC in DNA from biopsies and ecDNA shows promising potential for use as a marker of cancer initiation and progression [141]. 5hmC has been used as a marker for the progression of hepatocellular carcinoma and non-alcoholic liver disease [142]. The levels of 5hmC were determined in rat and human liver samples by using dot blot analysis and immunohistochemistry.

Circulating ecDNA biomarkers based on 5hmC are highly prognostic for colorectal cancer and gastric cancer and outperform traditional biomarkers [32]. Guler and colleagues demonstrated the non-invasive detection of pancreatic ductal adenocarcinoma by 5hmC alterations in ecDNA [143]. It has been demonstrated that differential hydroxymethylation is particularly indicative of genes associated with pancreatic development or function and cancer pathogenesis. By using nano-hmC-Seal [120], Tian and co-authors mapped and created 5hmC profiles in patients with esophageal cancer and identified reliable 5hmC signatures associated with this disease in ecDNA [144].

5hmC accumulates in the brain, with levels increasing significantly from the fetal stage to adulthood. The differential distribution of 5hmC in the brain and the fact that neurons are enriched in 5hmC approximately 10-fold compared with some peripheral tissues or embryonic stem cells suggest that 5hmC is a stable modification that may provide an epigenetic signature specific to neuronal function and/or brain dysfunction [145,146]. Whole-genome mapping studies of 5hmC in the cerebellum and hippocampus have revealed an enrichment of 5hmC in genes that are activated during development.

To date, there is evidence linking genome-wide perturbations mediated by 5hmC levels to diseases such as autism, Alzheimer’s disease, Huntington’s disease, Louis-Bar syndrome, and X-linked tremor/ataxia syndrome. The role of 5hmC in the pathogenesis of psychiatric disorders, including bipolar disorder, schizophrenia and clinical depression, has also been confirmed [147]. Although the precise molecular role of 5hmC is not fully understood, the fact that post-mitotic neurons are enriched in 5hmC suggests that 5hmC may influence the proper development of the nervous system by regulating the expression of genes responsible for the proliferation, development, and maintenance of neurons. The relationship between 5hmC levels and brain development, as well as the accumulation of 5hmC with age, leads researchers to consider this epigenetic modification a molecular marker of mental health: genes that acquire 5hmC with age are involved in intracellular cascades related to neurodegenerative diseases.

Depletion of 5hmC was observed in the hippocampus, cerebellum and entorhinal cortex of patients with Alzheimer’s disease. Conversely, enrichment of 5hmC in the frontal and middle temporal gyri was positively correlated with AD symptoms. These findings indicate that 5hmC may regulate key molecular components associated with Alzheimer’s disease progression in specific brain regions. Notably, 5hmC levels associated with Alzheimer’s disease can be detected in both preclinical and later stages of the disease, suggesting that 5hmC may serve as a viable biomarker for the onset and progression of Alzheimer’s disease [148].

Dong and colleagues demonstrated that patients with bipolar disorder and schizophrenia exhibit elevated levels of 5hmC and TET1 expression, but not TET2 and TET3, in the inferior parietal lobule [149]. The detection and quantification of 5mC and 5hmC were performed by using dot blot analysis. In contrast, patients with clinical depression showed a significant reduction in 5hmC levels [150], as determined by ELISA commercial quantification kits (MethylFlash™ methylated DNA and MethylFlash™ hydroxymethylated DNA kits). Min and colleagues, in 2022, investigated the whole-genome profiles of 5mC and 5hmC in the substantia nigra of patients with Parkinson’s disease [151]. The previously established chemical labeling and affinity purification method [135] was employed in combination with high-throughput sequencing (hMe-Seal) for 5hmC profiling and with (h)MeDIP-seq for 5mC profiling. A total of 4119 differentially hydroxymethylated regions were identified, and no methylated regions were observed in the postmortem brains of PD patients in comparison with controls. The hydroxymethylated regions exhibited specificity for patients with PD. A gene ontology analysis revealed that several pathways, such as neurogenesis and neuronal differentiation, were significantly enriched with 5hmC in the context of the disease.

As previously stated in the introduction, methods employed for the detection of 5hmC in DNA samples are often based on existing methods for the detection of 5mC. Some methods allow for the simultaneous detection of not only 5hmC, but also 5fC, 5caC and 5mC. While the potential regulatory roles of 5fC and 5caC are still being explored, obtaining information about both methylation and hydroxymethylation levels in DNA facilitates the comprehension of the mechanism underlying the relationship between epigenetic modifications and disease biology. First, the ratio of 5hmC to 5mC in the intragenic regions of genes is a better predictor of gene expression than any single marker alone. Second, 5hmC is oxidized from 5mC, suggesting that the interaction between 5hmC and 5mC may be critical for gene regulation [31].

The aforementioned facts support the consideration of the differential detection and mapping of 5hmC as a potential biomarker for mental and physiological health.

## 9. Conclusions

The biochemical mechanisms of 5hmC formation in DNA have been extensively studied (see Figure 1), and comprehensive genomic profiles of 5hmC distribution in a variety of tissues and cell lines have been established. 5hmC is not just an intermediate product of DNA demethylation; it possesses functional significance. In contrast to the conversion of cytosine into 5mC in DNA, the conversion into 5hmC has been demonstrated to result in transcriptional activation. This evidence suggests that 5hmC is a stable cytosine modification with its own role in the epigenetic regulation of the genome and gene expression [152].

A review of the methods used to detect DNA methylation has revealed that the traditional approach of sodium bisulfite treatment is unable to distinguish between the methylated and hydroxymethylated forms of cytosine. This suggests that numerous previous studies have reported a combined effect of both 5mC and 5hmC, with the results often attributed solely to 5mC. Consequently, any conclusions regarding the lack of diagnostic significance may be erroneous due to the research methodologies employed.

**Figure 17:**
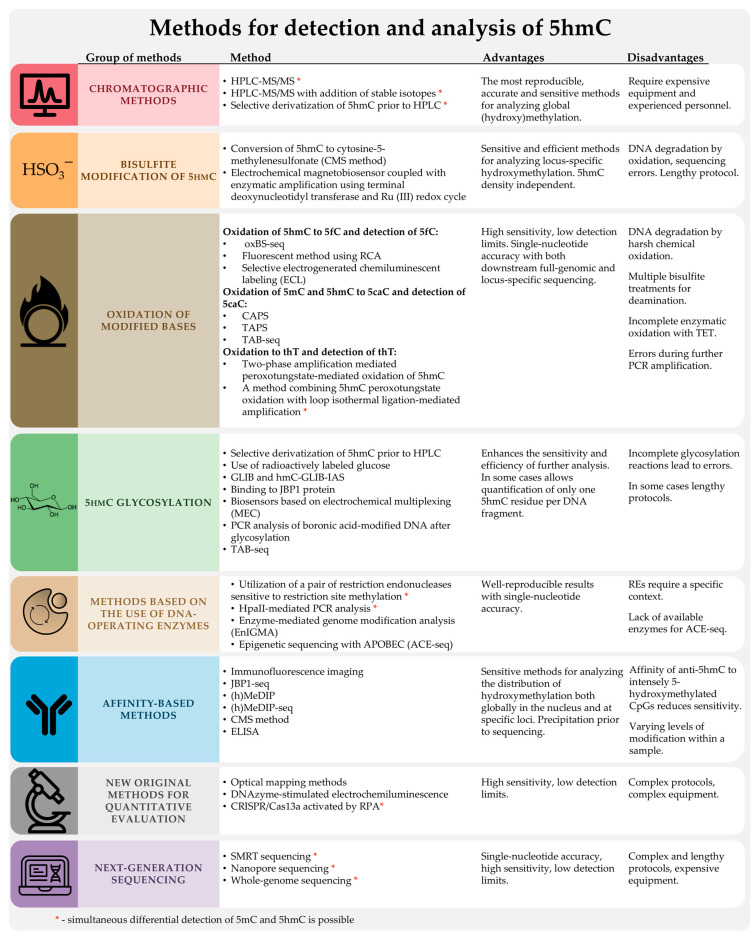
Methods for detection and analysis of 5-hydroxymethylcytosine. Some methods belong to more than one group. **Chromatographic methods:** HPLC-MS/MS [41,45,48], HPLC-MS/MS with stable isotope labeling [49,50] and selective derivatization of 5hmC prior to HPLC [54,55,56]. **Bisulfite modification of 5hmC:** CMS method [72,73] and electrochemical magnetobiosensor [83]. **Oxidation of modified bases:** oxBS-seq [76,80], fluorescent method using RCA [81], ECL [82], TAPS [84], CAPS [85], TAB-seq [40,74], peroxotungstate oxidation [89,90,91,93] and loop-mediated isothermal amplification [92]. **5hmC glycosylation:** selective derivatization of 5hmC prior to HPLC, use of radiolabeled glucose [59], GLIB and hmC-GLIB-IAS [66,67,68,69], binding to JBP1 protein (MBD-seq) [60,61], MEC biosensor [70], PCR analysis of boronic acid-modified DNA after glycosylation [71] and TAB-seq. **Methods based on the use of DNA-operating enzymes:** use of restriction endonucleases sensitive to DNA methylation [153], HpaII-mediated qPCR [97,100], EnIGMA [101] and ACE-seq [102]. **Affinity-based methods:** immunofluorescence imaging [104,106,107,108], JBP1-seq [65], (h)MeDIP and (h)MeDIP-seq [28,108,109,110], CMS and ELISA [105]. **New original methods for quantitative evaluation:** optical mapping [31,124], electrochemiluminescence [125] and CRISPR/Cas13a activated by RPA [126]. **Next-generation sequencing:** SMRT sequencing [112,113,114], nanopore sequencing [116,117,119] and whole-genome sequencing [120,122,123].

A comprehensive review of the major contemporary methods for the determination of 5hmC, together with an assessment of their respective merits and limitations, is presented in Figure 17. These methods are classified not only according to the chemical reactions involved but also from a biochemical perspective. Consequently, in some cases, a single method may fall into more than one category.

DNA methylation analysis is a valuable tool for understanding the progression of some diseases and is increasingly used as a diagnostic and prognostic clinical biomarker. Future studies could use advanced techniques, such as single-cell sequencing and CRISPR-based tools, to elucidate the role of 5hmC in gene regulation during disease progression. By identifying disease-specific 5hmC patterns, researchers can better understand how this modification affects cellular processes in diseases such as cancer and neurological disorders. Potential therapeutic strategies include the development of drugs that target the enzymes responsible for adding or removing 5hmC, such as TET enzymes, to restore normal epigenetic regulation. In addition, epigenetic editing tools such as CRISPR-dCas9 could be used to directly modify 5hmC marks at disease-relevant loci. The near future in the development of this field is seen as the discrimination of the effects of 5mC and 5hmC, which imposes requirements for methods of differential detection of various cytosine modifications and the need to map the positions of modifications. At present, this can be achieved through the use of SMRT and nanopore sequencing techniques. A significant challenge in methylation research is the lack of differential methods that can be employed simultaneously for all methylation modifications (5mC, 5hmC, 5fmC and 5caC) that are involved in the active demethylation of 5-methylcytosine (Figure 1). It is currently possible that the incorrect interpretations of the effects of methylation in the regulation of genome functioning may occur, which could be associated with misinterpretations of data. Following the development of 5fmC and 5caC basecalling models for raw-signal interpretation, both SMRT sequencing technology and nanopore sequencing have the potential to fulfil the aforementioned objective.

It is already evident that 5hmC plays a distinctive role in the pathogenesis of diverse neurological disorders, including neurodegenerative conditions. Despite numerous studies indicating a correlation between 5hmC abnormalities and psychiatric disorders, the precise molecular function of 5hmC remains elusive. Given that 5hmC is enriched in postmitotic neurons, it is hypothesized to be involved in the regulation of neuronal proliferation, development, and maintenance. The measurement of 5hmC levels could prove a valuable tool for the early diagnosis of cancer by considering 5hmC a marker of cell- or tissue-specific gene expression. However, the limitations of its detection methods must be taken into account when establishing the functional role of 5hmC.

The necessity to identify the epigenetic modification 5hmC has prompted researchers to develop a number of methods for qualitative and quantitative analysis in recent years. The development of 5hmC detection methods is still ongoing. In a review of methods for investigating epigenetic modifications in DNA [11,12,13], the authors anticipated that the oxBS-Seq and TAB-Seq strategies proposed the year before would significantly impact epigenetic DNA research. A decade later, it can be argued that these methods, along with the widely used HPLC, HPLC-MS and immunoassay-based detection, have become established in research practice and are now considered classics in 5hmC detection. The rapid advancement of technologies has facilitated significant progress in the study of the full-genomic distribution of 5hmC. This has led to the establishment of methods that involve the enrichment of genomic regions containing 5hmC, which can then be subjected to NGS and TGS.

This review provides a summary of both ‘old’ methods that have gained widespread use and those that have been proposed recently, within the past two years. Some of these methods permit the detection and tracking of the dynamics of not only 5hmC but also 5fC and 5aC, thereby facilitating a deeper understanding of the mechanisms underlying the etiology and pathogenesis of numerous diseases. It is conceivable that in 10 years’ time these methods will also become the ‘gold standard’ for detecting cytosine modifications in DNA. The analysis of methodologies for the detection of cytosine modifications indicates that only a limited number of methods, particularly third-generation sequencing, are capable of differentiating between 5hmC and other cytosine modifications, as well as determining the position of these modifications.

The ongoing development of chemical tools has significantly advanced the study of natural cytosine modifications, enabling more precise detection and mapping. To select the most suitable method for a given study, it is important to weigh the benefits and limitations of the available techniques, taking into account the study’s objectives and associated labor costs.
